# Multivariate unmixing approaches on Raman images of plant cell walls: new insights or overinterpretation of results?

**DOI:** 10.1186/s13007-018-0320-9

**Published:** 2018-07-04

**Authors:** Batirtze Prats-Mateu, Martin Felhofer, Anna de Juan, Notburga Gierlinger

**Affiliations:** 1 Department of Nanobiotechnology, BOKU-University of Natural Resources and Life Sciences, Muthgasse 11/II, 1190 Vienna, Austria; 2 Chemometrics Group, University of Barcelona, Diagonal 645, 08028 Barcelona, Spain; 3 Institute for Building Materials, Eidgenössische Technische Hochschule Zurich Hönggerberg, 8093 Zurich, Switzerland; 4 Applied Wood Research Laboratory, Empa-Swiss Federal Laboratories for Material Testing and Research, Überlandstrasse 129, 8600 Dübendorf, Switzerland

**Keywords:** Confocal Raman microscopy, hyperspectral imaging, vertex component analysis, non negative matrix factorization, multivariate curve resolution, plant cell wall, wood, Arabidopsis

## Abstract

**Background:**

Plant cell walls are nanocomposites based on cellulose microfibrils embedded in a matrix of polysaccharides and aromatic polymers. They are optimized for different functions (e.g. mechanical stability) by changing cell form, cell wall thickness and composition. To reveal the composition of plant tissues in a non-destructive way on the microscale, Raman imaging has become an important tool. Thousands of Raman spectra are acquired, each one being a spatially resolved molecular fingerprint of the plant cell wall. Nevertheless, due to the multicomponent nature of plant cell walls, many bands are overlapping and classical band integration approaches often not suitable for imaging. Multivariate data analysing approaches have a high potential as the whole wavenumber region of all thousands of spectra is analysed at once.

**Results:**

Three multivariate unmixing algorithms, vertex component analysis, non-negative matrix factorization and multivariate curve resolution–alternating least squares were applied to find the purest components within datasets acquired from micro-sections of spruce wood and Arabidopsis. With all three approaches different cell wall layers (including tiny S1 and S3 with 0.09–0.14 µm thickness) and cell contents were distinguished and endmember spectra with a good signal to noise ratio extracted. Baseline correction influences the results obtained in all methods as well as the way in which algorithm extracts components, i.e. prioritizing the extraction of positive endmembers by sequential orthogonal projections in VCA or performing a simultaneous extraction of non-negative components aiming at explaining the maximum variance in NMF and MCR-ALS. Other constraints applied (e.g. closure in VCA) or a previous principal component analysis filtering step in MCR-ALS also contribute to the differences obtained.

**Conclusions:**

VCA is recommended as a good preliminary approach, since it is fast, does not require setting many input parameters and the endmember spectra result in good approximations of the raw data. Yet the endmember spectra are more correlated and mixed than those retrieved by NMF and MCR-ALS methods. The latter two give the best model statistics (with lower lack of fit in the models), but care has to be taken about overestimating the rank as it can lead to artificial shapes due to peak splitting or inverted bands.
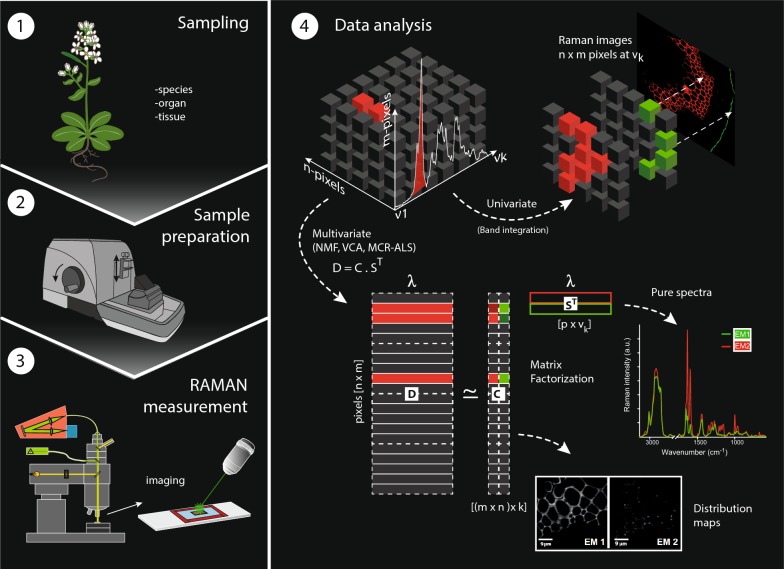

**Electronic supplementary material:**

The online version of this article (10.1186/s13007-018-0320-9) contains supplementary material, which is available to authorized users.

## Background

In the past years, Confocal Raman Microscopy (CRM) has gained great interest as characterization technique for biological materials due to the high lateral resolution [1]. Depending on the numerical aperture of the objective and the excitation wavelength of the laser, a CRM set up can reach a spatial resolution down to 250 nm [2, 3]. The advantages of CRM are many since one can acquire a chemical profile of the sample in a non-destructive and fast way, without time consuming sample preparation and staining. The coupling of rapid high sensitivity detectors and the xyz-driven piezo scan tables with high precision have allowed CRM to become suitable for Raman imaging i.e. chemical spatially resolved profiling. This is very important when analysing heterogeneous systems like biological samples.

Plant cell walls make the skeleton of the plant body and differ in their chemical and structural properties depending on species, age, environmental conditions and also position within the same tree/plant [4–9]. The potential of CRM has been shown in many studies by revealing the spatial distribution of lignin [10, 11] and other aromatic substances [12], the location of carbohydrates as pectin [13, 14], the orientation of cellulose in the cell wall [15], conformational changes of proteins [16], the accumulation of fats and waxes of the plant cuticle [12, 17] and also minerals [18, 19]. An extensive practical guide on Raman imaging of plant cell walls can be found in [20]. Specific band integration of different functional groups serves always as a first univariate approach and visualizes approximately component distributions [21]. In Raman spectra of plant cells, many of the bands are not sufficiently sharp and clearly separated and attributable to a specific functional group of only one plant cell wall component. Instead, they are often broad and overlapping. The overlapping bands of different components and the large amount of data in an image scan are often a limiting factor for classical univariate approaches and can be overcome using multivariate data analysis. Over the years, the most recurrent methods in multivariate image analysis have been Principal Component Analysis (PCA) [22] and cluster analysis (hierarchical or k-means clustering) [23]. PCA aims at the optimal description of the relevant variance in the original data set through a bilinear model based on principal components, i.e. uncorrelated variables calculated to capture the directions of maximum variance of the data set. PCA models allow performing a reduction in the dimensionality of the original data set, remove random noise and display relevant information about the pixel and spectral space of the image [24]. However, small variations that do not contribute to main fluctuations in the spectroscopic signal, e.g. different molecular structures of the same molecule, different orientations or different steps in a molecular pathway, can be eventually masked if an insufficient number of principal components is used. Cluster analysis by contrast is a segmentation algorithm oriented to find groups of pixels with similar spectra, i.e. with similar chemical composition, but finding the right number of clusters is also complex [25].

Spectral unmixing algorithms also describe images through a bilinear model analogous to the Beer–Lambert law [26], but they define the necessary components using “biological” meaningful constraints i.e. non-negativity and others, instead of orthogonality. The basic model of any spectral unmixing algorithm is described as follows:

1 $$ {\mathbf{D = CS}}^{{\mathbf{T}}} $$

where **D** (sized *m* × *n*) is the original data set that contains all the pixel (spectra) of the image, **C** is the matrix of concentration profiles or coefficients (sized *p * × *n*) and **S**^**T**^ is the named pure spectra, or dictionary matrix **(**sized *m* × *p* elements), that contains the profiles of the pure components as a result of the unmixing process. **C** contains the related concentration profiles (or abundances) of each pure spectral contribution (**S**^**T**^) in the different pixels. The denomination and characters used to describe the matrices in the bilinear model in Eq. 1 may change among unmixing methods, but not the underlying model. Each pixel spectrum in a hyperspectral image formed by positive spectral features is in all approaches explained by a linear combination of the pure endmember spectra forced to positive values (non-negativity constraint). They are weighted by a set of positive coefficients, which design the abundance of each pure endmember signature in the reconstruction of each particular pixel spectrum [27]. There are several unmixing methods applicable to spectroscopy but also to other fields [28, 29]. Among them, Vertex Component Analysis (VCA), Non-negative Matrix Factorization (NMF) and Multivariate Curve Resolution Alternating Least Squares (MCR-ALS) have become quite popular.

The VCA algorithm has been used in monitoring cellular uptake [30], in depicting the distribution of amygdalin in apricot seeds [31], in giving insights into the plant cell wall structure [32], in the selection of marker spectra of hyperspectral images of leafs in order to determine the pigment content [33] or in unmixing optoacoustic data [34]. VCA assumes that all pixels in the image are in a space (simplex of a determined order depending on the number of endmembers) defined by the purest components (vertices) called endmembers (EM) and that the affine transformation of a simplex is also a simplex. VCA has low computational complexity and needs the presence of pure pixels in the data to ensure the recovery of the correct endmembers. The data are projected orthogonally into the space given by the spanned endmembers. After each step, the extreme of the projection corresponds to the new endmember signature until the number of endmembers is exhausted. The sequential projections are made in such a way that the endmembers recovered are positive and the sum of abundances of compounds in every pixel equals one [35].

NMF has been successfully used in deciphering the complexity of samples in different fields including time resolved optical waveguide absorption spectroscopy [36], astronomical spectroscopy data [37], chemical agent detection by Raman spectroscopy [38], fluorescence spectroscopy [39], nuclear magnetic resonance data [40], text mining [41], facial recognition [42], gene expression analysis [43] and unsupervised audio-visual document structuring [44]. In addition, NMF has also shown its potential in resolving spectra of Raman images of the plant cell wall [45]. Non-negative matrix factorization (NMF) describes the original matrix of spectral data through a bilinear model of non-negative constituent factors [42]. Some NMF implementations contain the sparseness condition as an additional constraint [46, 47]. NMF is an iterative algorithm that combines the endmember spectra in order to reproduce the original data set as accurately as possible, i.e. providing models with an explained variance as high as possible. The number of components needed (or rank) is defined by the user trying to establish a compromise between good data approximation (high rank) and low model complexity (small rank). The quality of the approximation of the product of the decomposed matrices **C** and **S**^**T**^ to give the original matrix **D** can be monitored by a cost function which can be the Euclidean distance between the single elements of **D** and the reproduced elements by the **CS**^**T**^ model [42, 48]. In contrast to VCA, the optimization of all endmembers is made simultaneously. The implementation of the NMF algorithm used in this work can also handle the presence of missing values in **D**.

MCR-ALS is an unmixing algorithm used in many diverse fields, such as image analysis [27], environmental analysis [49], protein processes [50] and—omics sciences [51], but also on Raman data of plants [52]. MCR-ALS is an iterative algorithm that optimizes **C** and **S**^**T**^ in an alternating least-squares way under constraints that provide chemically meaningful profiles [53–55]. As for NMF, all components in the model are optimized simultaneously and the MCR model aims at describing the maximum variance of the original data set. The advantage of MCR-ALS is that multiple constraints can be added to the analysis. In image analysis, apart from non-negativity, the use of reference spectra, the incorporation of information of presence/absence of components in pixels (local rank constraints, [56]) and, recently, constraints that may improve the description of spatial patterns in maps [57, 58], can be taken into account. The number of components to be included in an MCR-ALS model can be decided by the user or be estimated by an auxiliary rank analysis method, such as Principal Component Analysis (PCA). The MCR-ALS optimization of **C** and **S**^**T**^ is most often controlled by comparing the data reproduced by the **CS**^**T**^ model with a noise-filtered matrix **D**, obtained from a PCA model using the same number of components as the MCR model. However, the use of this PCA-filtered matrix can be avoided and the MCR model can be optimized by comparing the **CS**^**T**^ model with the original experimental data. Usually, the PCA-filtering step is convenient because the removal of noise stabilizes the optimization process. However, in very few instances, where very minor components have to be retrieved or when the spectral overlap is extremely high among some components, the PCA filtering step may present the risk of excluding some relevant information that represents a very low percent of variance [59]. Hence, it may be interesting using the algorithm with and without incorporating the PCA filtering step. Furthermore, the implementation of multiset analysis is also an option when having several related data sets and allows for the simultaneous analysis of all information of interest [60]. In image analysis, MCR-ALS results can be used a posteriori as seeding information for other chemometric tools, such as segmentation methods, [61], calibration tasks [62] or super-resolution approaches [63].

In this manuscript we have investigated the applicability of the three different multivariate approaches, namely VCA, NMF and MCR-ALS (with and without PCA filtering) for unsupervised unmixing (resolution) of Raman spectra acquired from different plant tissues and species. Raman mappings of microsections of spruce wood (*Picea abies* L. Karst.) Spruce and *Arabidopsis thaliana* have been studied to include more homogenous tissues (wood) with secondary cell walls as well as different tissues and cell wall types (parenchyma, xylem), respectively. The aim was to compare the potential of the different algorithms, work out the influence of pre-processing (especially baseline correction) and the effect of the selection of other algorithm-specific input parameters.

## Methods

### Plant material and microsectioning

**Fig. 1:**
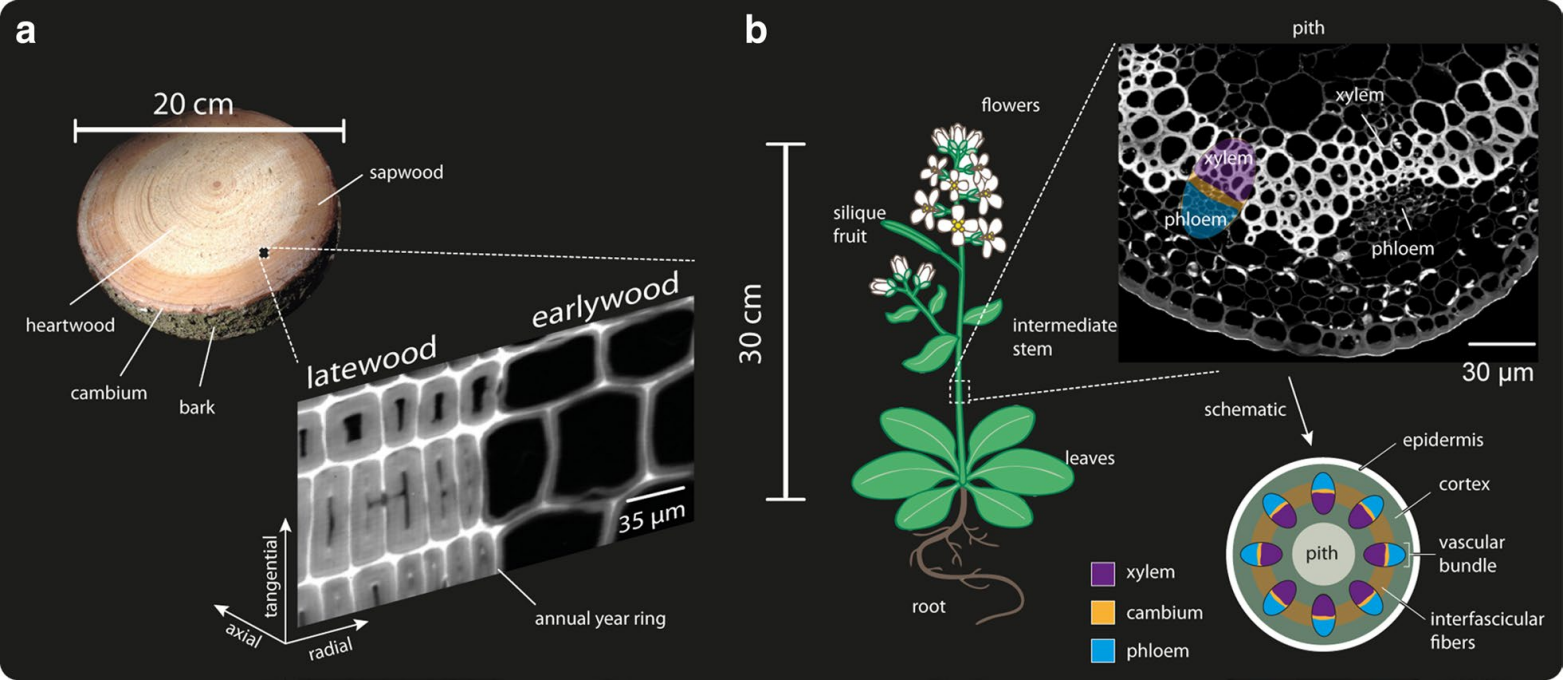
Anatomy of the samples from which the Raman datasets were generated. **a** Left: Disk cut from the stem of Spruce. Right: The zoom in the stem is based on the CH stretching integration Raman image of an annual ring with early—(EW) and latewood (LW). **b** Left: Schema of the grown plant *Arabidopsis thaliana*. On the right part, a CH stretching integration Raman image of a microsection of the stem is shown and the main anatomical parts are pointed out: xylem, cambium, phloem and pith

A 90 years old Spruce tree (*Picea abies* (L.) Karst.) was harvested in the middle of July 2015 in Mühlviertel (Upper Austria). The bark of the fresh stems was removed immediately to avoid diffusion of bark compounds into the cambium and wood cells, which may enhance burning of the sample during the Raman measurement (personal observation). Pieces were cut out of a stem disc from 130 cm above ground (breast height) comprising the transition zone between sapwood and heartwood (see Fig. 1a). The blocks were trimmed and cut in 20 µm thick cross sections using a rotary microtome (RM2235, Leica Biosystems Nussloch Gmbh, Germany). The stem of a 30 cm tall wild type *Arabidopsis thaliana* (see Fig. 1b) was embedded in polyethylenglycol (PEG 2000, Sigma Aldrich, Austria) following the protocol described in [20] and cut in 3 µm thick sections. Afterwards, the embedding media was washed out thoroughly with Millipore water. The microsections were put on glass slides with a drop of water, covered with glass coverslips (0.17 mm thick) and sealed with nail polish, to avoid water evaporation during the Raman experiment.

### Confocal Raman microscopy

Raman spectra from the native cross sections were acquired using a confocal Raman microscope (alpha300RA, WITec GmbH, Germany) with a 100 × oil immersion objective (numerical aperture (NA) = 1.4, coverslip correction 0.17 mm) (Carl Zeiss, Germany). The samples were excited with a linear polarized (0°) coherent compass sapphire green laser λ_ex_ = 532 nm (WITec, Germany). The scattered Raman signal was collected with an optic multifiber (50 µm diameter) to a spectrometer (UHTS 300 WITec, Germany) (600 g mm^−1^ grating, spectral resolution about 3.8 cm^−1^, maximum and minimum error of about 4.8 and 2.9 cm^−1^, respectively) and finally detected by the CCD camera (Andor DU401 BV, Belfast, North Ireland). The maximum spatial resolution is given by r = 0.61λ/NA, which for the parameters and confocal set up used is about 230 nm. The Control Four (WITec, Germany) acquisition software was used for the Raman imaging set up. For both samples, the laser power was set at 36 mW. For Spruce, an integration time of 0.13 s was used and one spectrum was taken every 0.5 µm. For *A. thaliana* one spectrum was recorded every 0.3 µm and the integration time set to 0.18 s.

### Data analysis

#### Spectral pre-processing

Data preprocessing was performed using the WITec Project Plus 4.0 software (WITec, Germany). Cosmic ray removal was carried out before any further analysis based on an intensity threshold set by taking into account spectral and spatial pixels adjacent to the pixel of interest. All spectra were cut to the spectral region from 300 to 1800 cm^−1^ before background subtraction and multivariate data analysis.

In order to assess the influence of the background subtraction on the unmixing algorithm output, analyses were done for both datasets with and without previous background correction (based on fitting a polynomial of order 3 and performed in the WITec Project Plus 4.0 software). For Spruce, a rank of 4 endmembers with and without background subtraction was taken for all methods, whereas when 5EM were applied, only the comparison for background subtracted data is shown. For Arabidopsis, the results are discussed with and without background subtraction based on the VCA analysis. The comparison of all approaches on the Arabidopsis dataset is based on the data without background subtraction.

#### Unmixing algorithms

During the last 4 years more than 10 different spruce and Arabidopsis samples have been measured and analysed beside many other different plant species. Thereof two Raman images have been chosen as the most representative and illustrative to show and verify the observed trends in performance of the algorithms and effects of pre-processing and number of endmembers.

All algorithms were tested with different number of endmembers and with and without background subtraction. The algorithm VCA was applied using the software Cytospec (v.2.00.01). Non-negative matrix factorization (NMF) was carried out using the WITecPlus 4.1 Software. The number of iterations was selected based on the retrieval of stable results, being for both datasets a minimum of 100,000 iterations. Multivariate Curve Resolution-package GUI 2.0 for MatLab (MathWorks, USA) was used for all MCR-ALS analyses [60]. In order to keep the parameters between methods as similar as possible, for MCR-ALS, non-negativity was used as a constraint in the concentration and spectral direction. Spectral signatures in **S**^**T**^ were normalized according to the Euclidean norm. The effect of a prior PCA filtering step was tested in the final MCR-ALS results for both data sets.

To compare analogous endmembers between different methods they were sorted according to the abundance map and endmember spectra similarity. Additionally, for control, the correlation coefficients between the endmember spectra given by VCA and all the endmembers of the other three methods were calculated.

#### Statistical data analysis

For all methods and conditions, the lack of fit (LOF) of the model and the variance explained (r^2^) by the model was calculated as done in [64]. The expressions used are described below:

2 $$ {\text{LOF }}\left( \% \right) = 100 \times \sqrt {\frac{{\mathop \sum \nolimits_{i,j} e_{i,j}^{2} }}{{\mathop \sum \nolimits_{i,j} d_{i,j}^{2} }}} $$

3 $$ r^{2} \left( \% \right) = 100 \times \left( {1 - \frac{{\mathop \sum \nolimits_{i,j} e_{ij}^{2} }}{{\mathop \sum \nolimits_{i,j} d_{ij}^{2} }}} \right) $$

where *d*_*ij*_ is the element of the original data matrix in row *i* and column *j* and *e*_*ij*_ is the residual obtained from the difference between the element *d*_*ij*_ of the original data set and the analogous element reproduced using the suitable unmixing model.

The Pearson correlation coefficient between pairs of spectral endmembers was calculated within a method to ensure that the correct number of endmembers was used (too high correlation coefficients could imply an unnecessarily high number of components). Correlation coefficients were also calculated between endmembers obtained with different unmixing methods to facilitate the intermethod endmember correspondence. The statistical analysis was conducted under MatLab environment (MathWorks, USA).

## Results

### Comparison of unmixing methods for Raman image analysis of spruce wood

**Fig. 2:**
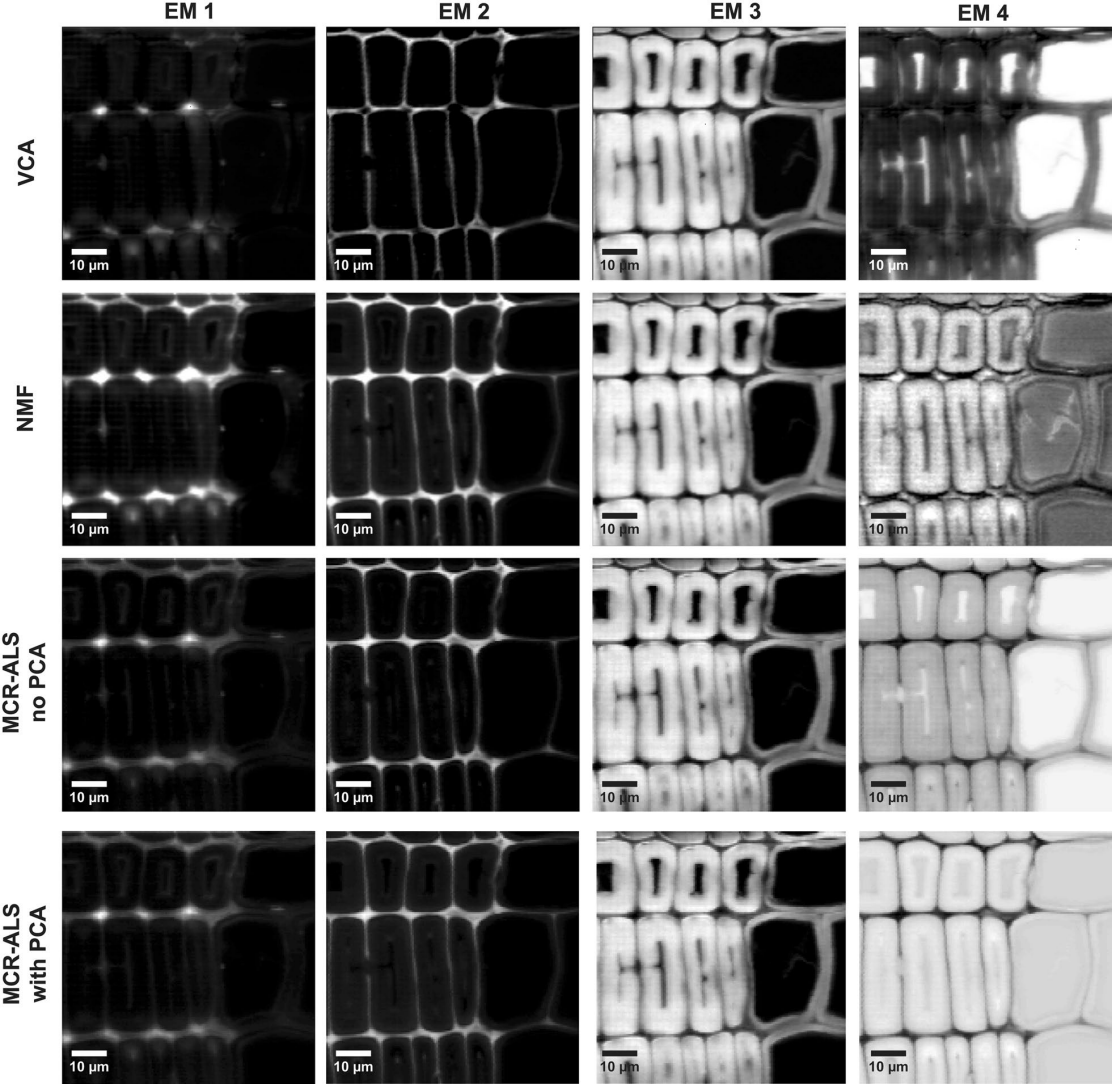
Unmixing methods on early and latewood of Spruce without background subtraction and based on 4 endmembers. Distribution maps given by all methods: from top to bottom VCA, NMF, MCR-ALS with no previous PCA and MCR-ALS with prior PCA. The images are scaled from minimum to maximum in each endmember. Black corresponds to the minimum intensity whereas white corresponds to the maximum

**Fig. 3:**
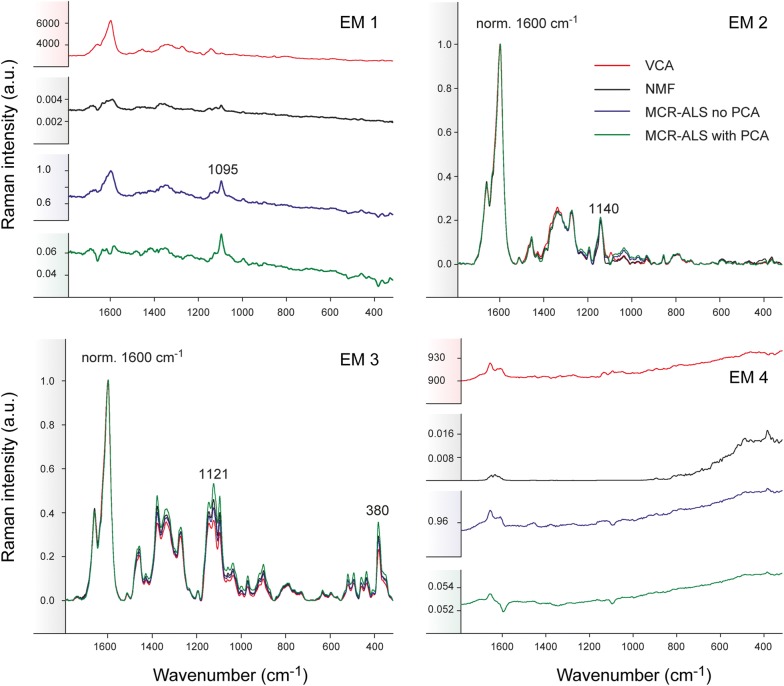
Comparison of multivariate methods on early and latewood of Spruce without previous background subtraction based on 4 endmembers. Endmember spectral signatures corresponding to the intensity maps shown in Fig. 2: VCA (in red), NMF (in black), MCR-ALS without PCA (in blue) and MCR-ALS with PCA (in green). The spectra are normalized against the main aromatic band at 1600 cm^−1^ except for EM1 and EM4

A Raman image of Spruce, comprising earlywood and latewood (Fig. 1a), was chosen to perform a first assessment of the performance of the three different multivariate methods on blind spectral resolution. All algorithms were first compared based on the results of non-background corrected spectra and 4 endmembers (EM) in the bilinear model (Figs. 2, 3). The abundance maps given by all methods (Fig. 2) recompose the wood tissue as the zones between the cells (cell corners and compound middle lamella with EM1 and EM2, respectively), the cell wall (EM3) and the cell lumen and/or cell wall (EM4). EM2 and EM3 are virtually identical for the three methods [abundances (Fig. 2) and spectra (Fig. 3)]. EM2 spectra reflect almost pure lignin (e.g. bands at 1600, 1660 and 1140 cm^−1^) [65–67] between the cells and EM3 a mixture of lignin (see bands before) and polysaccharides (e.g. bands at 1121, 1095 and 380 cm^−1^ [68]), in the cell wall (Fig. 3). EM1 and EM4 are more different between the three methods. EM1 includes only the cell corner region in VCA (while in EM2 more compound middle lamella, Fig. 2, first row), and the endmember spectrum reflects again clear lignin bands (Fig. 3, EM1 red spectrum). The 4th endmember clearly reflects the lumen (Fig. 2). So VCA abundance maps reflect the most different regions compared to the other methods (Fig. 2), although correlation coefficients between endmember spectra are high (Additional file 1: Table S1A). For all other three methods these two endmembers are present at more than one region, EM1 in cell corner and compound middle lamella and EM4 in lumen and cell wall (Fig. 2). The distinction of the compound middle lamella in x-direction by the other methods in EM1 is reflected in the spectra by a band around 1095 cm^−1^ (Fig. 3), known to be sensitive for cellulose orientation and high in the cell wall layer S1 and compound middle lamella in laser polarisation direction [15]. EM4 abundance maps include in all approaches the lumen, although in different scale (white to dark grey) but in NMF and MCR approaches also the secondary cell wall (Fig. 2). All EM4 spectra have low intensity spectra and in MCR-ALS also intensity minima, which appear like inverted bands. This is reflected in a high negative correlation with EM1 (Additional file 1: Table S1A). In the case of NMF, neither the abundance map nor the spectrum are interpretable for EM4. Therefore, it seems that for NMF and MCR-ALS approaches either the rank is still too low or a baseline correction is needed to clarify the results, although the models explain 99.99% of variance and have a low lack of fit (0.92–3%) (Table 1). On the contrary VCA model statistics are inferior, but abundance maps and endmember spectra are sound and interpretable.

**Table 1:** Summary of the data analyses performed on both data sets

Sample	Method	BG subtraction	No. of EMs	LOF (%)	Variance explained (%)
Spruce	VCA	No	4	13.1280	98.2766
	NMF	No	4	3.0743	99.9055
	MCR-ALS				
	Without PCA	No	4	0.92782	99.9914
	With PCA	No	4	0.92107	99.9915
	VCA	Yes	4	35.5641	87.3946
	NMF	Yes	4	6.5643	99.5691
	MCR-ALS				
	Without PCA	Yes	4	6.247	99.6098
	With PCA	Yes	4	6.2243	99.6126
	VCA	Yes	5	38.7925	84.9514
	NMF	Yes	5	6.5691	99.5685
	MCR-ALS				
	Without PCA	Yes	5	5.2069	99.7289
	With PCA	Yes	5	5.2136	99.7282
Arabidopsis	VCA	No	6	33.9914	88.44
	NMF	No	6	13.2726	98.2384
	MCR-ALS				
	Without PCA	No	6	0.6314	99.9960
	With PCA		6	0.6239	99.9961

### Influence of the background subtraction on the algorithm performance

**Fig. 4:**
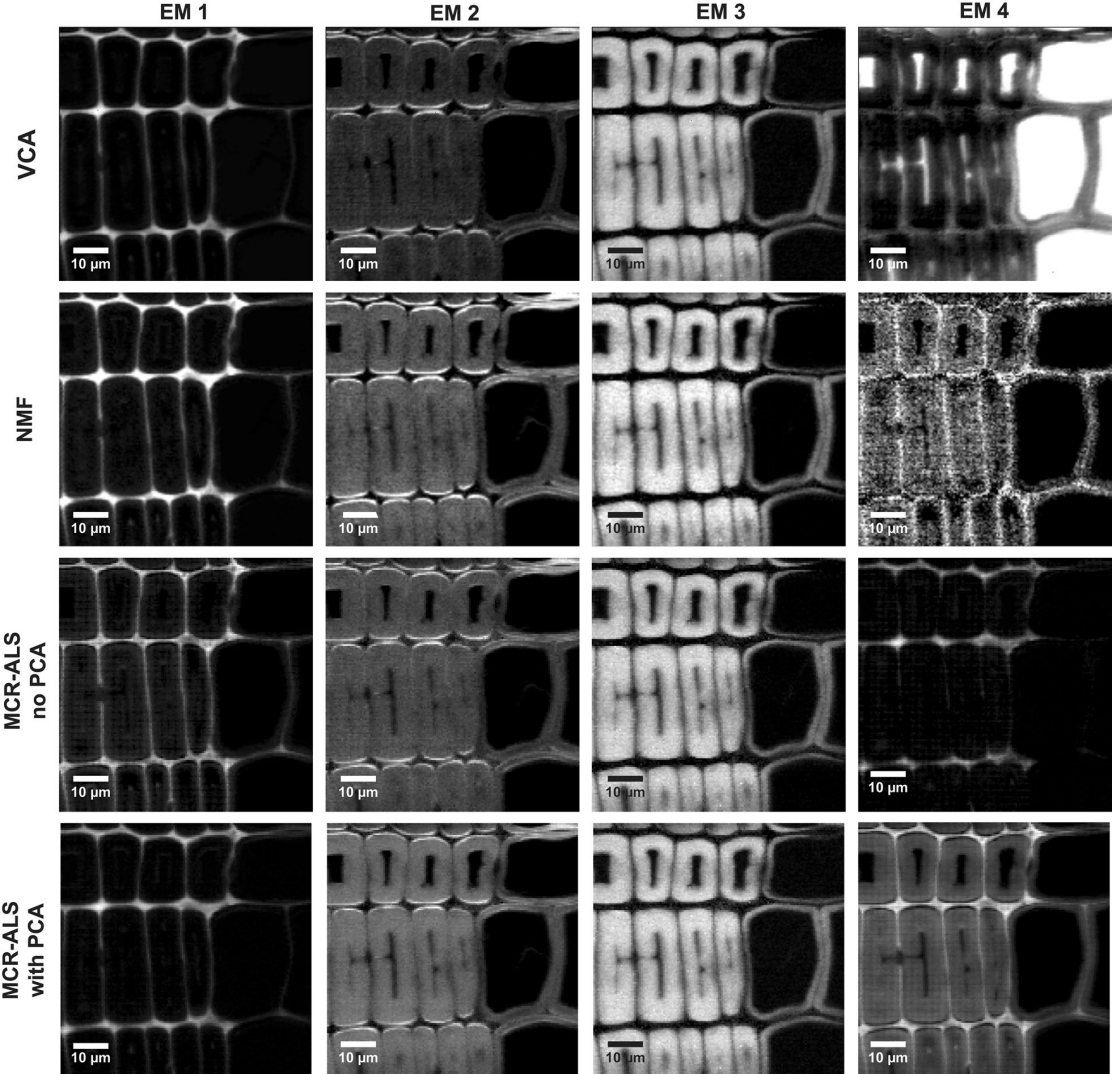
Comparison of abundance maps given by VCA, NMF, MCR-ALS without PCA and MCR-ALS with PCA on background subtracted Raman data of Spruce based on 4 endmembers

**Fig. 5:**
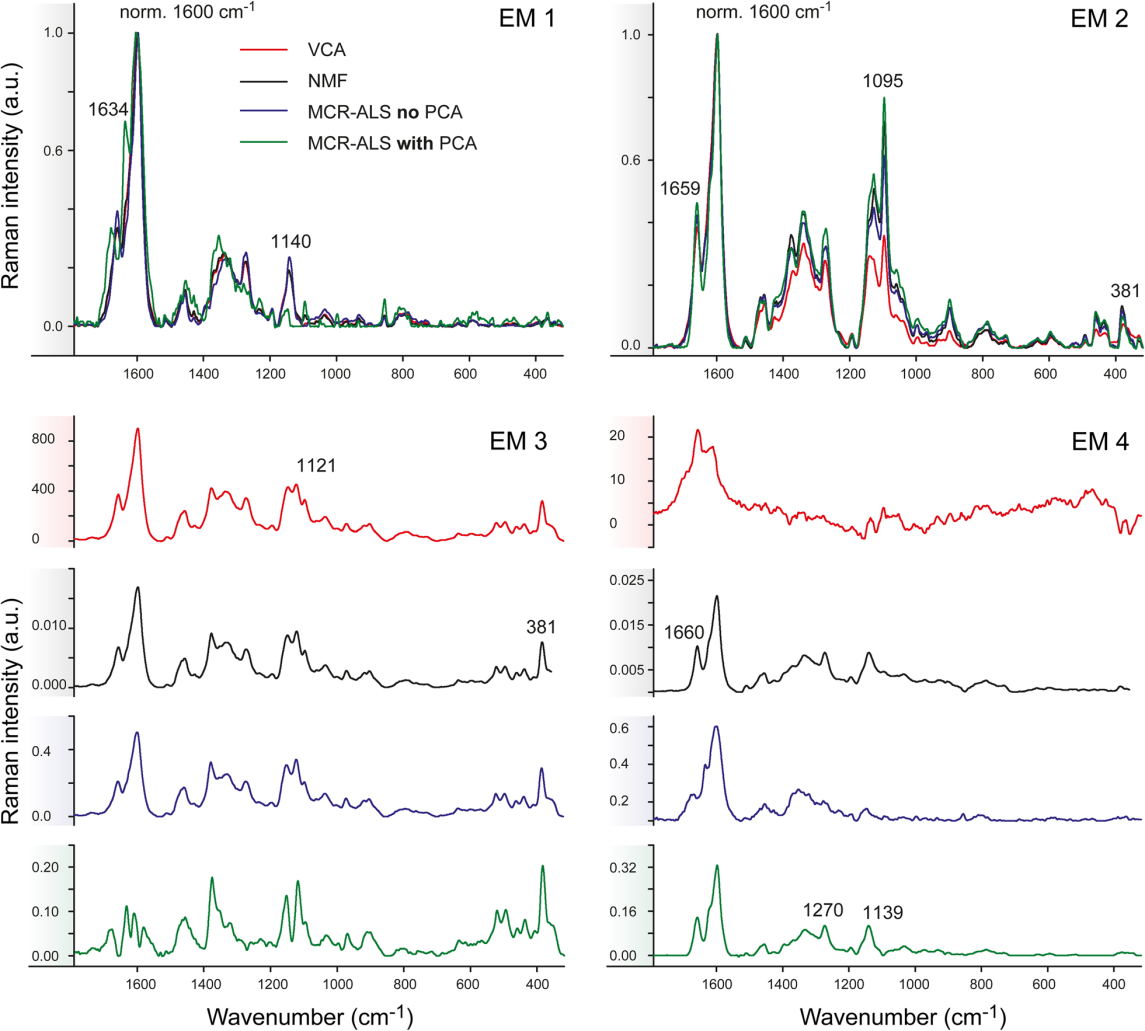
Comparison of multivariate methods on background subtracted Raman data of Spruce. Endmember spectra given by VCA (in red), NMF (in black), MCR-ALS without PCA (in blue) and MCR-ALS with PCA (in green). Endmembers are grouped following the column order given by Fig. 4

The effect of background subtraction was examined by applying all methods on the background subtracted Spruce dataset and keeping the rest of the parameters constant i.e. same number of endmembers (4 EM), number of iterations (100,000) or convergence criterion (Figs. 4, 5). All algorithms delivered three very similar components: cell corner together with compound middle lamella (EM1), the S1 and S3 cell wall layers parallel to the laser polarization direction (EM2), showing the microfibrils oriented with high angle with respect to the fibre axis, and the main cell wall layer S2 (EM3) (Fig. 4). VCA brought the lumen in the 4th endmember, albeit the rest of algorithms did not. So once again, VCA behaves most different from the others in (1) resulting in the most different abundance maps and (2) a clear endmember for the water filled lumen. The other three methods traced in EM4 the compound middle lamella and/or cell corners (Fig. 4) with typical lignin spectra (Fig. 5). EM4 is strongly correlated with EM1 using the NMF and MCR approaches (Additional file 1: Table S1B), as both endmembers represent lignin, but of different composition/structure (Fig. 5).

In general, the correlation coefficients between endmembers within each method increase significantly after background correction, although MCR with PCA preserves a higher difference (Additional file 1: Table S1B). Unfortunately, this higher difference is also reflected in “artificial” not meaningful spectra (green spectrum, EM3, Fig. 5), where neither the lignin nor the cellulose bands are recovered in their typical form.

The comparison with (Figs. 4, 5) and without baseline correction (Figs. 2, 3) clearly showed that removing the background eases to reveal differences in cellulose structure. The cell wall layers with high cellulose microfibril angle (S1) are described by one endmember after background subtraction (Figs. 4 and 5, EM2). While the VCA abundance map displayed the S1 selectively, all others methods resulted in medium (grey) values in the S2 layer as well. At the same time changes in the aromatic composition are more difficult to track and overseen by VCA. In a next step the number of endmembers was increased to 5 to explore the potential if subtle compositional changes in cell wall composition could still be revealed.

### Effect of the number of endmembers on the algorithm output

**Fig. 6:**
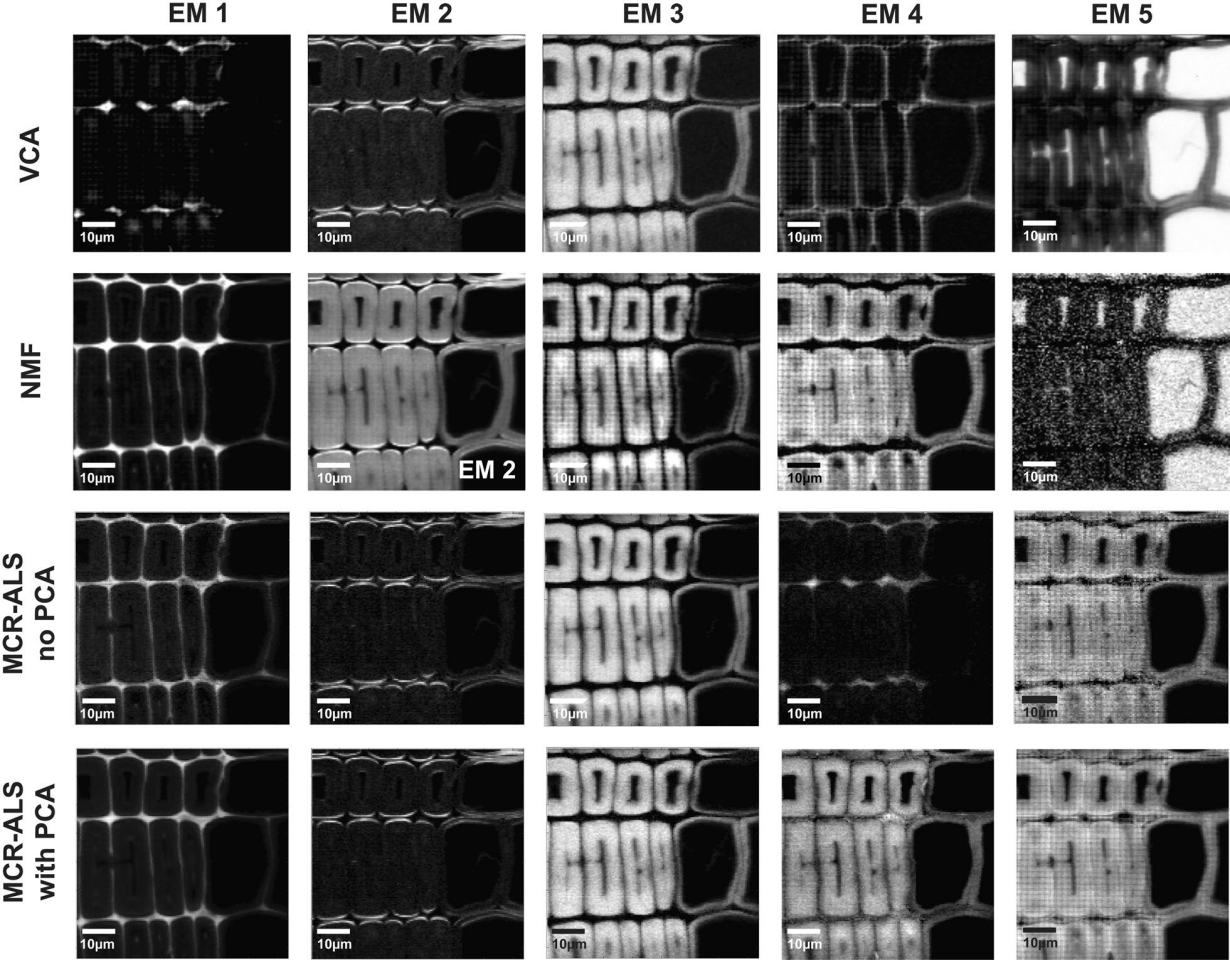
Comparison of multivariate methods on Raman data of Spruce with 5 endmembers and previous background subtraction. Distribution maps given by all methods: from top to bottom VCA, NMF, MCR-ALS with no previous PCA and MCR-ALS with prior PCA

**Fig. 7:**
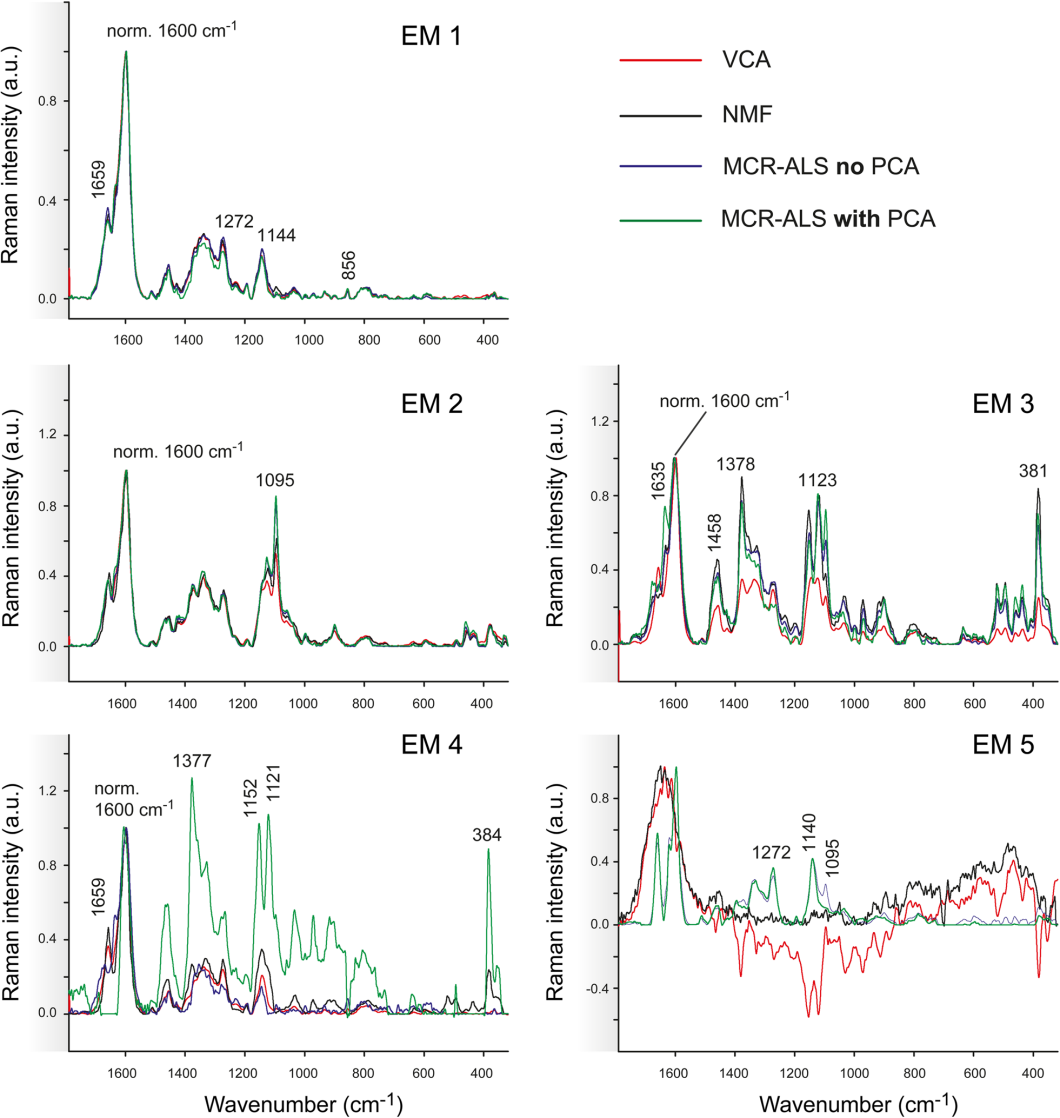
Comparison of endmember spectra given by VCA (in red), NMF (in black), MCR-ALS without PCA (in blue) and MCR-ALS with PCA (in green) of Raman data of Spruce with 5 endmembers and previous background subtraction

The dataset Spruce was analysed by all three approaches with 5 initial endmembers and after baseline correction (Figs. 6, 7). The correlation coefficients within the methods for the case are displayed in Additional file 2. The first three endmembers (EM1, EM2 and EM3) are again distinguishing the three most different wood tissue parts (Fig. 6). Especially the first two endmember spectra become now very similar in the three approaches, and differences are mainly found for EM3, EM4 and EM5 (Fig. 7). EM4 is rather inconsistent in relation to the pixel intensity distribution (Fig. 6), but also to the endmember spectral shape (Fig. 7). VCA pictures mainly middle lamella (cell corner earlywood) with a lignin-like endmember spectrum in Fig. 7. NMF pictures a patchy cell wall with higher intensity at tangential and radial cell junctions and is composed of lignin in its majority and polysaccharides in minor amounts. MCR-ALS without PCA remits the cell corners (Fig. 6, EM4) with other type of lignin composition (shoulder at 1635 cm^−1^) (see Fig. 7, EM4). The most dissonant endmember is given by MCR-ALS with prior PCA (Fig. 6) with both the cell wall S2 and partially the cell corners and again a very “artificial” not interpretable spectrum (Fig. 7). As the related endmember obtained with no filtering is very minor in presence, it seems that such a contribution may have disappeared after PCA filtering. The last endmember, EM5, portrays the water filled lumen for VCA and NMF (Fig. 6), whereas by both MCR-ALS variants the secondary cell wall is displayed again. The related spectra are matched also two by two: VCA and NMF deliver a Raman signature of water and both MCR-ALS analysis carry spectra with cell wall bands, but again in an unusual “artificial” manner. Model statistics (Table 1) did only improve slightly for NMF and MCR-ALS approaches and got worse for VCA, although this approach gave once again the most interpretable results and spectra.

### Performance on chemically more heterogeneous plant cell walls

All algorithms were additionally tested on a more heterogeneous plant sample, a cross section of an *Arabidopsis thaliana* stem (named Arabidopsis) whose anatomy is described in Fig. 1B. The recorded Raman image comprised different cell wall types: lignified xylem cells, cambium and phloem with primary cell walls. Again, different conditions (baseline correction, number of endmembers) have been tested, mainly by the fast VCA approach. Up to 6 endmembers, relevant pattern and spectra have been revealed and again an influence of background subtraction detected (see Additional file 3). Without background subtraction, the middle lamella and cell corners are better defined (EM1) (Additional file 3: Fig. S1A). The second endmember shows pointwise distribution in the cell corner close to the cambial zone (EM2) with a distinct lignin band at 1634 cm^−1^ (Additional file 3: Fig. S1C, red spectrum), pointing to different lignin composition during cell wall formation. After background correction (Additional file 3: Fig. S1B) this differentiation is less clear and the distribution patterns (EM1, EM2) show stripe-wise artefacts. For these reasons, no background correction was selected for comparison of the three approaches (VCA, NMF, MCR-ALS) on Arabidopsis. The rest of the endmembers are in general very similar and describe carbohydrates (EM3, EM4), proteins and lipids (EM5) and the lumen (EM6). Thus also in this dataset the influence of background subtraction is observed mainly for endmembers involving aromatic cell wall components.

**Fig. 8:**
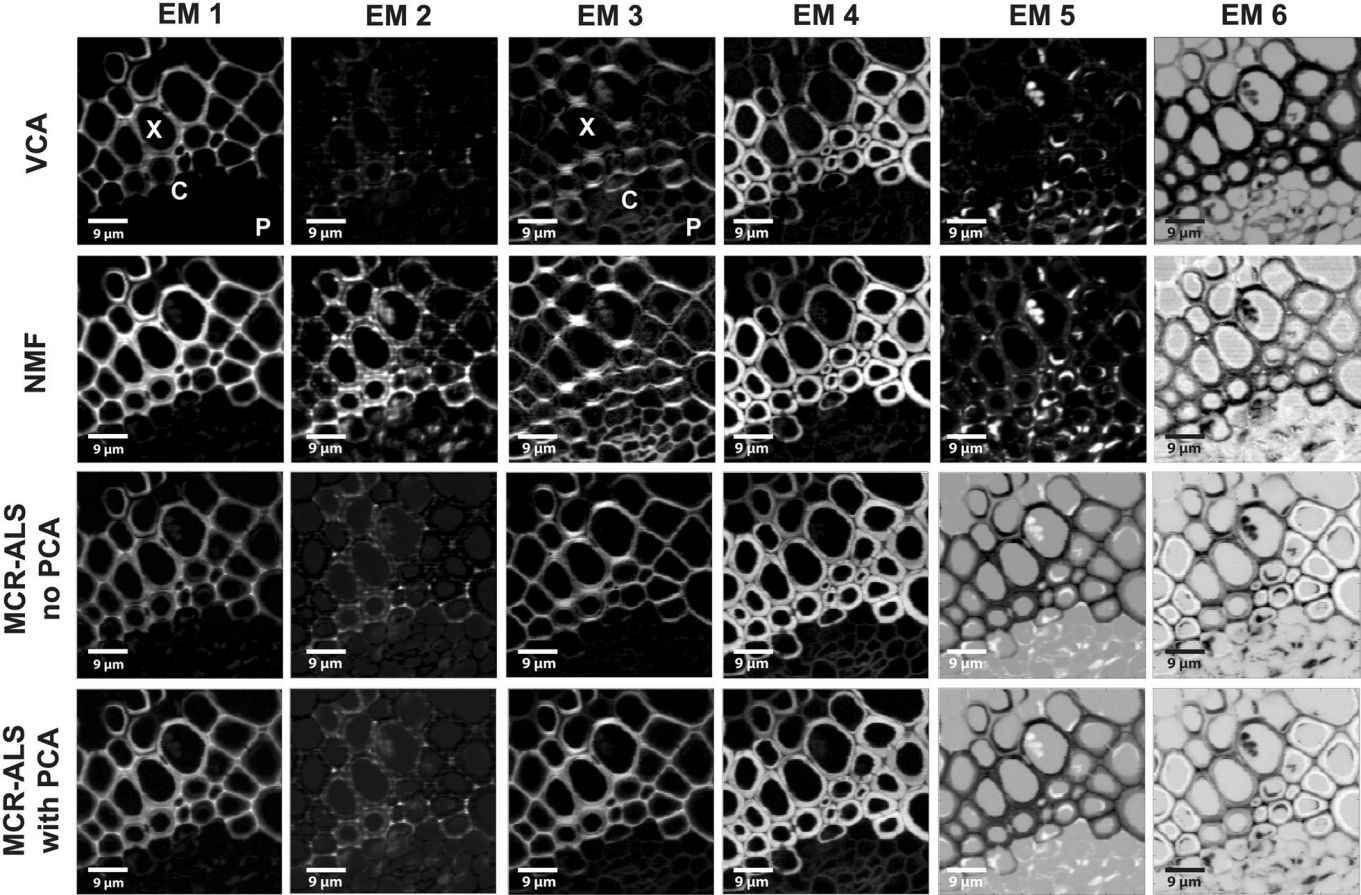
Comparison of multivariate methods on raw Raman data of Arabidopsis without previous baseline correction. Abundance maps given by VCA, NMF, MCR-ALS without PCA and MCR-ALS with PCA. *X* xylem, *C* cambium and *P* phloem

**Fig. 9:**
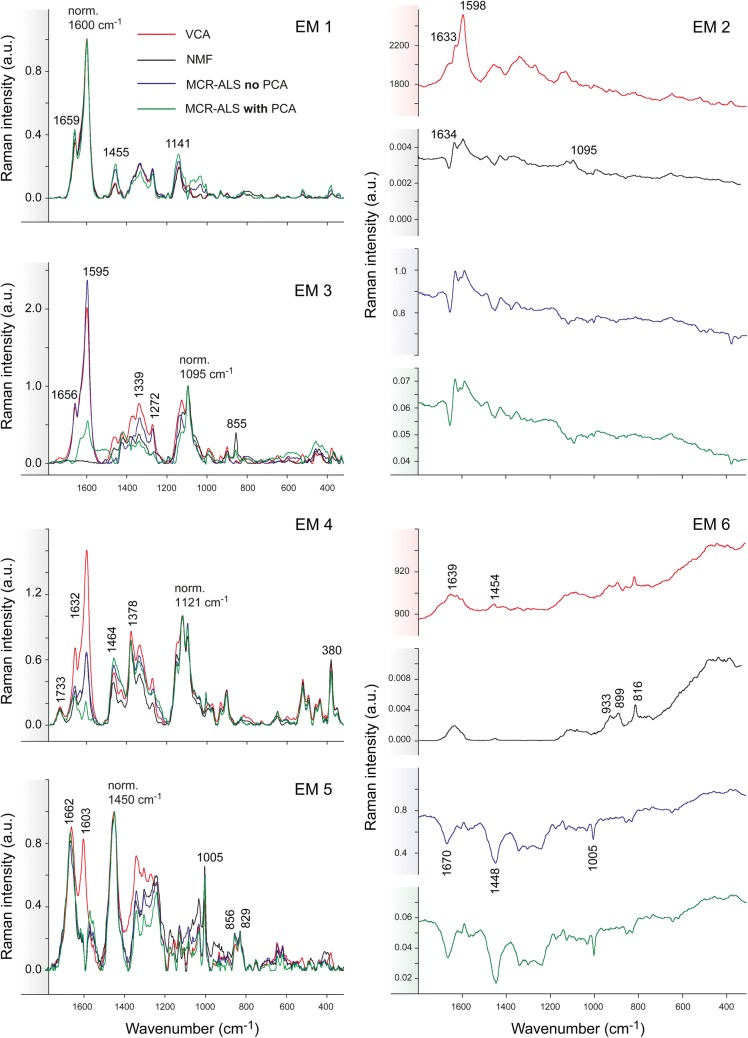
Comparison of multivariate methods on raw Raman data of Arabidopsis. The endmember spectra were baseline corrected and each endmember group was normalised against the most intense band (EM1: 1600, EM2: 1600, EM3: 1095, EM4: 1122 and EM5: 1458 cm^−1^) except for the EM6 due to the lack of a common most intense band

Figures 8 and 9 show the comparison of all abundance maps and their corresponding endmember spectra, respectively, given by the four methods for Arabidopsis without background correction. The different approaches result in similar abundance maps, especially EM1 and EM4 representing the xylem cell corner and compound middle lamella (EM1) and cell wall (EM4). Differences are observed for the other EMs; this time especially NMF is most different from the rest of the methods. NMF yields for EM2 a combination of cell corners and cell wall with a pointwise distribution of the highest intensity, whereas for all the remaining three analyses only the cell corners near the cambial zone are emphasized (Fig. 8). Yet all approaches agree in finding a 1633 cm^−1^ band (Fig. 9, EM2), which is assigned to C=C of coniferyl alcohol and C=O of coniferyl aldehyde aldehydes in the lignin structure [65, 67, 69, 70]. While only a shoulder with VCA, the other two (NMF and MCR) end up in a sharp band. NMF shows also cellulose bands (1122, 1094 cm^−1^) in accordance with the highlighted cell wall in the abundance map. In addition, NMF diverges in the abundance map of EM3, where beside the enhancement of the S1 wall of the xylem, also the primary cell wall (cambium and phloem) is highlighted together with the S1 cell wall layer of the xylem. The corresponding EM3 spectrum shows beside the characteristic cellulose orientation sensitive band at 1095 cm^−1^, a clear pectin signal at 855 cm^−1^ for the α-1,4-glycosidic bond [71] and no contribution in the aromatic regions (black spectrum), whereas the others did. In EM4 the aromatic contribution was different between the approaches [high 1600 cm^−1^ band for VCA, medium for NMF and MCR without PCA, no aromatics and almost pure cellulose for MCR with PCA, (Fig. 9)], although the abundance maps are similar for all methods (Fig. 8). EM5 presents in all methods material attached to the cell wall, mainly in the phloem part (Fig. 8). The EM5 spectra coincide well between all methods (Fig. 9) with bands assigned to proteins and lipids (amide III band at 1666 cm^−1^, CH_2_ and asymmetric CH_3_ bending at 1450 cm^−1^ and phenylalanine at 1005 cm^−1^) [72–74]. VCA (in red) is the only one having also the aromatic band at 1600 cm^−1^. In EM6 all methods highlight the lumen, but NMF also the primary cell wall. The two MCR approaches additionally depict further cell wall features i.e. the thick cell wall layer S2 of the interfascicular fibers (in white, Fig. 8).

Correlation coefficients between spectral endmembers (see Additional file 4) within the same method are lower than for data set Spruce (see Additional files 1 and 2), ratifying the higher heterogeneity of the sample Arabidopsis. The lowest values are again for MCR with previous PCA, followed by NMF. In the same manner as for data set Spruce, VCA presents the highest correlation values between endmembers. This confirms the finding of the endmember spectra, where VCA showed more mixtures of the different components, whereas NMF and MCR resulted in more “pure” component spectra.

For all constraints and methods, the correlation coefficient between each VCA and the rest of endmembers given by the other algorithms was calculated prior any pre-processing, and is shown in Additional files 5, 6 and 7 for Spruce with 4EM (with and without baseline correction), Spruce with 5EM (with background correction) and Arabidopsis with 6EM (with background subtraction), respectively. The highest correlations match the endmember assignments.

## Discussion

Plant cell walls are highly variable biological materials, which differ in their structure and composition to fulfil different requirements in the living plant [75, 76]. In order to assess the suitability of multivariate unmixing methods for the resolution of Raman imaging of plant material in a more general case, data have been acquired on different cell walls of two species. Wooden secondary cell walls of a spruce tree as well as a stem section of *Arabidopsis thaliana* have been analysed by multivariate analysis approaches. The stem area measured in spruce comprises the thin walled earlywood cells optimized for water transport as well as the thick walled latewood cells, which give the tree mechanical support (Fig. 1A). Between these two functional tissues, no differences have been revealed based on the acquired spectra. The stem of *Arabidopsis thaliana* includes different cell wall types: the phloem (living cells for transport of solutes from the leaves to growing tissues), cambium (dividing cells) and the xylem (dead cells for water transport from the root to the leaves) (Fig. 1B), which showed clear differences based on the Raman images. These generated images of plant tissues are well known, which facilitates the interpretation of the data, and also offer a great example for the application of multivariate methods due to the high correlation at the topochemical level (pixels far away might be related) but also at the chemical level (pixel spectra are highly correlated).

Multivariate spectral unmixing analysis describes the input Raman images as a combination of concentration maps and spectral Raman signatures or endmembers (chemical composition) based on several premises and constraints, which might differ to pursue the most appropriate resolution. A summary of all analyses performed and their lack of fit and variance explained by the model is given in Table 1. In all cases better statistics (high explained variance, low lack of fit) are achieved by MCR-ALS and NMF approaches compared to VCA. VCA works sequentially and assuming the presence of pure endmembers, unlike NMF or MCR-ALS, which optimize the endmembers simultaneously, balancing the correlation between endmembers and seeking for the highest variance. This simultaneous extraction helps in obtaining components with less correlation among them with the latter two (see Additional files 1, 2 and 4). MCR-ALS with PCA filtering delivered often the least correlated endmembers, but sometimes spectral signatures exhibited inverted meaningless bands in the endmember spectra (e.g. Figure 3, EM1 and EM4, in green). The results have shown the risk of PCA filtering when small variance contributions are of interest (high rank), since these can be lost in the discarded noise-related principal components.

### The choice of rank

The approximate number of endmembers can be determined by taking into account the previous knowledge on the inner variability in the sample (known components) but also by performing singular value decomposition, which factorizes the original data matrix and delivers the eigenvectors, ordered following the amount of variance explained. Since small variance contributions could be mistaken as noise contributions, it is advisable using the unmixing methods with a variable number of components to investigate whether also minor contributions can be modelled in a meaningful way.

For Spruce, different numbers of endmembers (2, 3, 4 and 5 EMs) were tested for VCA and NMF (see Additional files 8, 9 and 10, and Figs. 2, 3). The results differed mostly at low rank values and is again a consequence of the different way of extracting components: VCA prioritizes extracting the positive endmembers, obtained by sequential orthogonal projections, whereas NMF or MCR-ALS extract simultaneously all components and seek explaining the maximum variance of the data set using a pre-set model size. Therefore, the latter two methods tend to extract preferentially high signal contributions that may account for a higher percentage of variance explained when the rank is too low. This explains that using 2 or 3 endmembers NMF did not include an endmember for the lumen (water), which is a contribution with very low signal intensity, but very different in spectral shape from the rest, while VCA extracted it (see Additional files 8 and 9, top). With higher rank (4, 5, 6) the most severe differences between the methods are always found for the low intensity endmember spectra (Figs. 4, 5, 6, 7, 8, 9). VCA always gives the most correlated endmembers (Additional files 1, 2 and 4), but all bands in endmember spectra are biological meaningful, even with high rank. In contrast, the other methods (especially MCR with PCA) may sometimes result in less interpretable spectral shapes in high rank endmember spectra, although often the best model statistics (uncorrelated EM, high explained variance and low lack of fit) are achieved (Table 1).

### Background subtraction: a matter of data set and research question

Multivariate analysis of spectroscopic data is influenced by the preprocessing strategy, especially baseline correction. As mentioned before, background subtraction might induce the loss of small features in the data if background differences are related to compositional changes. On the other hand, relevant signal features can be enhanced and differentiation among contributions might become easier after background subtraction. Which side weights more is a matter of method, data set and research question.

The results given by VCA were quite similar between non- and background subtracted data, for ranks of 2 and 3 endmembers (Additional files 8 and 9, top). For the same rank, NMF shows more differences among background and non-background corrected data (Additional files 8 and 9, bottom), again because of the different way of extracting components. While VCA always has one endmember describing the lumen as most different, NMF reveals already more detailed changes in chemistry and structure. With 3 endmember it is clearly seen that without baseline correction EM2 describes the cell corner with different aromatic structure (Additional file 9: Fig. S3A), while with background subtraction a change in cellulose microfibril angle is revealed in the S1 layer (Additional file 9: Fig. S3B). This trend of emphasizing either aromatic differences without baseline correction or changes in cellulose microfibril angle after baseline correction is clearly confirmed by all three approaches when 4 endmembers are used for calculation (compare Figs. 2 and 3 with Figs. 4 and 5). The distinct lignin component (Figs. 2, 3, EM1) found when no background subtraction was applied was replaced by the radial cell wall layer S1 (Figs. 4, 5, EM2 with cellulose microfibrils parallel to the laser polarization) after the analyses on baseline corrected data. Certainly, the prior EM1 and EM2 (Figs. 2, 3) was enclosed in the current EM1 as seen by the presence of the pectin band at 856 cm^−1^ (Figs. 4, 5), which might indicate that a rank of 5EM could be useful for this particular differentiation. Despite background subtraction, VCA was again the single algorithm handing the water in lumen as an endmember (Figs. 2, 3, EM4) because there is still a residual contribution of this component with a sufficiently distinct shape. For NMF and MCR-ALS this may not happen because the variance due to background contribution is almost totally removed and the methods focus on capturing spectral variations linked to higher percentages of variance. Thus, the 4^th^ endmember did not become alike after pre-processing, but showed for NMF and both MCR-ALS features of the middle lamella with lignin bands at 1140, 1270, 1600 and 1660 cm^−1^. The ratio between the bands at 1600 and 1660 cm^−1^ of EM4 changed between methods, indicating a different resolution of the ratio of C=C in lignin [77]. MCR-ALS without previous PCA was able to resolve both two types of lignin and the S1 radial cell wall layer and its spectral signature was the most different with the highest 1600/1660 ratio and the additional presence of the shoulder at 1634 cm^−1^ (Fig. 5, EM4, in blue).

The correlation between endmembers within the same method increased and the values were more akin amidst methods [Additional file 1: Table S1A (not baseline corrected) and 1B (with prior baseline correction)]. This also clearly shows that variability has been removed by background subtraction. The background subtraction affected differently each method in terms of lack of fit. The lack of fit of the model given by VCA (rank = 4EM) increased when background subtraction was carried out (Table 1) because the algorithm still sought the lumen contribution, very different in shape to the rest and now with very low variance. For NMF, the lack of fit decreased substantially, indicating a better performance when background subtraction was applied. For NMF and both MCR-ALS variants, the lack of fit was generally lower because these methods seek the explanation of the maximum variance with a predefined model size.

For Arabidopsis, the background correction had not so much influence on the resulting endmembers, although this dataset showed more types of cell walls and thus more variability. The VCA analysis with and without background subtraction (Additional file 3) resulted in similar abundance maps and spectra using 6 endmembers. After baseline correction some abundance maps showed a striped pattern (especially EM1 and EM2), probably an effect of the noise of the CCD camera [78]. Furthermore, without baseline the cell corners were selectively spotted near the cambial zone, whereas after baseline correction also other tissues have been included. This showed that cell corners near the cambial zone had differed composition (spectral band shoulder at 1634 cm^−1^) (Additional file 3: Fig. S1C red spectrum), not seen after baseline correction (Additional file 3: Fig. S1D, red spectrum). Based on this information and due to the stripes no previous background subtraction was selected for the final analysis and comparison among methods (Figs. 8, 9).

### Holding hands: optimizing rank according to background subtraction

The results on data set Spruce clearly showed that the rank has to be adjusted according to the algorithm and/or background correction. While VCA needed a 5th endmember to resolve the change in aromatics and microfibril orientation, the other approaches revealed these differences after baseline correction already with 4 endmember (Fig. 4). This is due to the already discussed fact that VCA always attributes one endmember to describe the watery lumen, while the others do not (details see above). If no baseline correction is done (Fig. 4) it becomes absolutely necessary for all methods to use 5EM, if additionally, the change in cellulose microfibril angle in the S1 layer is of interest. In the case of Spruce, for VCA, 5EM with prior background subtraction gave the best results. For the rest of the methods, 4EM with previous background subtraction were enough since the background signal is taken as an offset or in the residuals of the algorithm.

All analysis showed that the performance of the different methods is dependent on the number of endmembers and the pre-processing strategy and it has to be adapted to every algorithm.

### Unmixing: interpretable meaningful results?

All unmixing methods revealed in both data sets almost pure lignin spectra from the cell corners and compound middle lamella in-between the cells. Similar like in previous VCA-studies on wooden cell wall [32], additional changes in lignin composition have been revealed, which would be overseen by simple band integration approaches. Cellulose came not as a pure endmember in Spruce, as no pure pixel has been found in this dataset. The analysis of poplar tension wood with Raman microscopy and MCR-ALS [52] could reveal a pure cellulose endmember, as the G-layer with pure cellulose was present. They suggested to work with four endmembers and discarded the rest as not unique. In our 2nd example on Arabidopsis the 6 endmembers delivered by VCA are optimal without baseline correction, as abundance maps display very different anatomical regions and all endmember spectra are interpretable. In all the analysis done so far, the VCA approach always gave the most interpretable spectra, but with the tendency to include more mixtures of different components. NMF and MCR-ALS approaches seem to retrieve the purest (less correlated) EMs, but sometimes include less expected band shapes. Especially MCR-ALS with PCA filtering has more risk of not retrieving subtle differences even choosing more endmembers because of the possible discard of useful information. In these cases, bands less easily interpretable may show up (e.g. Fig. 5: green spectrum EM3, Fig. 7: green spectrum EM4, Fig. 9: green spectrum EM3, EM6). The thin cambial and phloem cells with low Raman intensity are in this example best retrieved by NMF in EM3: clearly visualized in the abundance maps (Fig. 8) and with a pectin band in the EM3 spectrum (Fig. 9). Nevertheless, a pure pectin spectrum was not revealed in this data set with the current settings, as it is mixed with cellulose in the S1 (high microfibril angle). Analysis of the epidermis of Arabidopsis in a previous VCA analysis revealed clearly a very pure pectin spectrum [12]. On contrary NMF analysis of the cambial cells walls of a carrot root retrieved cellulose together with pectin [45]. By applying a sparse NMF version, which incorporates graph relationship to overcome overlapping problems, spectra of pectin, cellulose and lignin were unmixed from a Raman image of a Longjing tea cell [46]. However, in the latter study, very artificial endmember spectra were found that did not match Raman spectra of reference substances in the literature [65, 66, 68, 71].

### Different results explained by different working procedures of the unmixing algorithms

With all four unmixing approaches the main trends in plant tissues are found consistently. As discussed above, differences arise in minor or very similar components, due to the factors driving the extraction of components in the different approaches and the general calculated statistics [correlation of endmember (r), lack of fit, explained variances] may vary as well.

The main differences in the working procedure of the algorithms are:

- The sequential optimization of components in VCA based on a series of orthogonal projections versus the global optimization by NMF and MCR-ALS based on achieving a maximum of variance explained. This makes that NMF and MCR provide generally an overall better description of the image data, with higher variance explained, and a major differentiation of all compounds since they are optimized simultaneously. VCA instead can sometimes point out very small variance contributions linked to components with very distinct spectrum that would need models with higher rank to be found.
- The assumption of closure (sum of abundances equal to 1) in VCA, which is needed for the optimization, is absent in NMF and MCR-ALS. The assumption of this constraint in VCA is an additional element that makes the model fit worse than for NMF and MCR in similar conditions, since a constraint not obeyed by the system is forced to obtain the final solutions.
- The use of a PCA step to set the experimental space in VCA and to do optionally noise-filtering in MCR-ALS, as opposed to NMF and MCR-ALS with no PCA filtering step. This PCA step is, in itself, not questionable, but may have different effects depending on the data set. As a general rule, the PCA step is beneficial since it implies a reduction of noise and helps to a better definition of the experimental space in VCA and a more stable least-squares optimization in MCR. However, it should be taken into account the risk that very subtle spectral differences or very minor contributions may occasionally be sent to the discarded ‘noise-related’ components and may not be recovered in the final unmixing results. In the case of MCR, an advisable practice is running the algorithm with and without the PCA filtering step to check for the presence of this risk. Or even better, applying more powerful constraints and work, whenever possible, in multiset analysis mode.

In terms of computation time, VCA is slightly faster than MCR-ALS and no choice of constraints or other tuneable parameters has to be performed. This makes it a suitable method to perform a quick exploration of data sets and may help to select rank. NMF has a computation time significantly higher than MCR-ALS but provides stable solutions if a sufficient number of iterations is allowed. MCR-ALS instead has a reasonable computation time and may allow for accommodating many other powerful constraints other than non-negativity that could help in a better differentiation of similar biological components.

## Conclusions

Raman spectroscopic imaging combined with multivariate data analysis gives detailed insights into plant cell wall design. The Raman spectra can be used to differentiate regions different in chemistry as well as in structural organization (cellulose microfibril angle). Even subtle changes in composition and structure are visualized and based on the pure component (endmember) spectra and the related maps, the heterogeneous plant structure can be properly described. In addition, unmixing of Raman plant images offers a step ahead in the definition of completely new tissues, cell or cell layer identification and classification.

All unmixing methods yield spectral vectors that exhibit signal-to-noise ratios vastly better than those of individual spectra and that describe different components (e.g. lignin, pectin) and/or anatomical regions (e.g. cell wall layers S1, S2). They do not rely on previous knowledge and personal decisions of selecting areas or thresholds for calculating average spectra to describe different plant tissues. Nevertheless, the effect of baseline correction and the number of endmembers have to be taken into account and selected according to the data set, research question and unmixing algorithm. The background signal can be in some cases advantageous since different chemical components generate a different background and, therefore, it can be useful to better differentiate similar components. On the contrary, when background is very dominant, this contribution may hinder the differentiation of subtle spectroscopic features in other relevant bands. Furthermore, the number of components to be resolved should be adapted accordingly in order to obtain the best description of the sample. While for VCA and NMF no baseline correction and up to 6 endmembers revealed interpretable interesting results in the examples presented, MCR-ALS approaches in Raman data of plant cell walls worked better with baseline corrected data and a smaller number of endmembers to describe the data.

## Additional files

**Additional file 1: Table S1.** Correlation coefficients between endmembers of each algorithm (VCA, NMF, MCR without previous PCA and with previous PCA) based on 4 endmembers of Spruce (A) without and (B) with prior background (BG) subtraction, ordered in descending order.

**Additional file 2: Table S2.** Correlation coefficients between the spectral endmembers generated by the algorithms (with 5 endmembers) for Spruce with previous background subtraction.

**Additional file 3: Figure S1.** Influence of background subtraction on the Vertex Component Analysis with 6 endmembers on *Arabidopsis thaliana*. Intensity maps given by VCA on xylem and phloem of A. thaliana (A) without previous background (A) subtraction and (B) after background subtraction. Abundance maps are scaled equally two by two. The respective endmember spectra are shown in (C) and (D).

**Additional file 4: Table S3.** Correlation coefficients between the spectral endmembers generated within each algorithm. (with 6 endmembers) for Arabidopsis without previous background subtraction.

**Additional file 5: Table S4.** Correlation coefficients between the spectral endmembers generated by the algorithms (4 endmembers) for Spruce without previous background subtraction. The endmembers given by VCA were taken as reference for the comparison.

**Additional file 6: Table S5.** Correlation coefficients between the spectral endmembers generated by the algorithms (5 endmembers) for Spruce with previous background subtraction. The endmembers given by VCA were taken as reference for the comparison.

**Additional file 7: Table S6.** Correlation coefficients between the spectral endmembers generated by the algorithms (6 endmembers) for Arabidopsis without previous background subtraction. The endmembers given by VCA were taken as reference for the comparison.

**Additional file 8: Figure S2.** VCA (top) and NMF (bottom) analyses of the data set Spruce without previous background subtraction (A) and with background subtraction (B) with a rank of 2 endmembers. VCA is able to separate the plant material (EM1) and water (EM2) independently of the implementation of background correction. However, the cell wall is included in the water endmember when no baseline correction is applied. NMF by contrary deprecates the water component and the plant material is described by compound middle lamella (EM1) and cell wall (EM2). A rank of 2 is not enough to depict the main features of the plant tissue even after background correction, as NMF does.

**Additional file 9: Figure S3.** VCA (top) and NMF (bottom) analyses of the data set Spruce, without previous background subtraction (A) and with background subtraction (B) with a rank of 3 endmembers.

**Additional file 10: Figure S4.** VCA (top) and NMF (bottom) analyses of the data set Spruce, without previous background subtraction (A) and with background subtraction (B) with a rank of 5 endmembers. For VCA (top), when no background subtraction is applied (A), EM1 shows the inner part of the cell corners whereas EM4 shows the outer cell corners but also part of the main cell wall. EM2 shows the cell wall layer S1 in which the cellulose microfibril orientation is parallel to the laser polarisation. Note the presence of pectin at 854 cm^−1^ in the cell corner in the EM1 and EM4. EM3 marks most of the cell wall (cellulose, hemicellulose and lignin) while EM5 is the lumen filled with water. (B) Distribution maps of the endmembers (EM) generated by VCA of Spruce with prior background subtraction. The abundance maps are similar distributed as (A) but for EM4, which does not incorporate parts of the cell wall but rather only the compound middle lamella. The intensity profiles of the same endmembers in A and B are equally scaled between same endmembers, having the brightest pixel the maximum intensity. (C) and (D) Corresponding characteristic endmember spectra of the abundance maps shown in (A) and (B), respectively. The main differences between EMs are attributed to the 1658 band (C=O stretching and C=C groups) (see also inserts) and the orientation of the cellulose microfibrils (bands at 1096, 1125 and lower spectral region 370–550 cm^−1^).

## Abbreviations

EM: endmember
BG: background
LOF: lack of fit
MCR-ALS: multivariate curve resolution-asymmetric least squares
NMF: non-negative matrix factorization
PCA: principal component analysis
VCA: vertex component analysis
