# Supplementary material for: Multivariate unmixing approaches on Raman images of plant cell walls: new insights or overinterpretation of results?
Source: Plant Methods. 2018 Jul 4;14:52. doi: 10.1186/s13007-018-0320-9 (PMC6031114; doi:10.1186/s13007-018-0320-9)
Supplement: Supplementary file 1 — Additional file 1: Table S1. Correlation coefficients between endmembers of each algorithm (VCA, NMF, MCR without previous PCA and with previous PCA) based on 4 endmembers of Spruce (A) without and (B) with prior background (BG) subtraction, ordered in descending order. [file 13007_2018_320_MOESM1_ESM.docx]

**Table S1**

| A) Without BG subtraction | | | | | | | | |
| --- | --- | --- | --- | --- | --- | --- | --- | --- |
| VCA | | **NMF** | | **MCR no PCA** | | **MCR with PCA** | | |
| EM | r | EM | r | EM | r | EM | | r |
| 1-2 | 0.985 | 2-3 | 0.836 | 2-3 | 0.838 | 2-3 | 0.765 | |
| 2-3 | 0.880 | 1-2 | 0.687 | 1-2 | 0.752 | 1-2 | 0.427 | |
| 1-3 | 0.820 | 1-3 | 0.583 | 1-3 | 0.656 | 1-3 | 0.342 | |
| 3-4 | -0.169 | 3-4 | -0.171 | 3-4 | -0.214 | 3-4 | | -0.358 |
| 2-4 | -0.381 | 2-4 | -0.317 | 2-4 | -0.334 | 2-4 | | -0.4857 |
| 1-4 | -0.519 | 1-4 | -0.772 | 1-4 | -0.816 | 1-4 | | -0.948 |

| B) With BG subtraction | | | | | | | |
| --- | --- | --- | --- | --- | --- | --- | --- |
| VCA | | **NMF** | | **MCR no PCA** | | **MCR with PCA** | |
| EM | r | EM | r | EM | r | EM | r |
| 2-3 | 0.951 | 2-3 | 0.930 | 1-4 | 0.961 | 1-4 | 0.855 |
| 1-2 | 0.920 | 1-4 | 0.924 | 2-3 | 0.912 | 2-4 | 0.783 |
| 1-3 | 0.852 | 2-4 | 0.918 | 1-2 | 0.813 | 1-2 | 0.580 |
| 1-4 | 0.538 | 3-4 | 0.916 | 2-4 | 0.767 | 2-3 | 0.384 |
| 2-4 | 0.369 | 1-3 | 0.837 | 1-3 | 0.767 | 1-3 | 0.240 |
| 3-4 | 0.253 | 1-2 | 0.796 | 3-4 | 0.732 | 3-4 | 0.210 |
