# Supplementary material for: Multivariate unmixing approaches on Raman images of plant cell walls: new insights or overinterpretation of results?
Source: Plant Methods. 2018 Jul 4;14:52. doi: 10.1186/s13007-018-0320-9 (PMC6031114; doi:10.1186/s13007-018-0320-9)
Supplement: Supplementary file 2 — Additional file 2: Table S2. Correlation coefficients between the spectral endmembers generated by the algorithms (with 5 endmembers) for Spruce with previous background subtraction. [file 13007_2018_320_MOESM2_ESM.docx]

**Table S2**

| With BG Subtraction | | | | | | | |
| --- | --- | --- | --- | --- | --- | --- | --- |
| VCA | | **NMF** | | **MCR no PCA** | | **MCR with PCA** | |
| EM | **r** | **EM** | **r** | **EM** | **r** | **EM** | **r** |
| 1-4 | 0.997 | 1-4 | 0.947 | 1-4 | 0.975 | 1-5 | 0.878 |
| 2-3 | 0.970 | 2-4 | 0.897 | 2-5 | 0.921 | 2-3 | 0.875 |
| 3-4 | 0.930 | 1-2 | 0.877 | 1-5 | 0.912 | 2-5 | 0.850 |
| 1-3 | 0.918 | 3-4 | 0.851 | 2-3 | 0.885 | 1-2 | 0.802 |
| 2-4 | 0.908 | 2-3 | 0.817 | 3-5 | 0.841 | 1-3 | 0.712 |
| 1-2 | 0.897 | 1-3 | 0.726 | 4-5 | 0.826 | 3-5 | 0.705 |
| 1-5 | 0.530 | 1-5 | 0.529 | 1-2 | 0.825 | 3-4 | 0.701 |
| 4-5 | 0.516 | 4-5 | 0.429 | 2-4 | 0.763 | 2-4 | 0.617 |
| 2-5 | 0.514 | 2-5 | 0.332 | 1-3 | 0.752 | 4-5 | 0.588 |
| 3-5 | 0.456 | 3-5 | 0.239 | 3-4 | 0.681 | 1-4 | 0.494 |
