# Supplementary figures and images for: Multivariate unmixing approaches on Raman images of plant cell walls: new insights or overinterpretation of results?
Source: Plant Methods. 2018 Jul 4;14:52. doi: 10.1186/s13007-018-0320-9 (PMC6031114; doi:10.1186/s13007-018-0320-9)

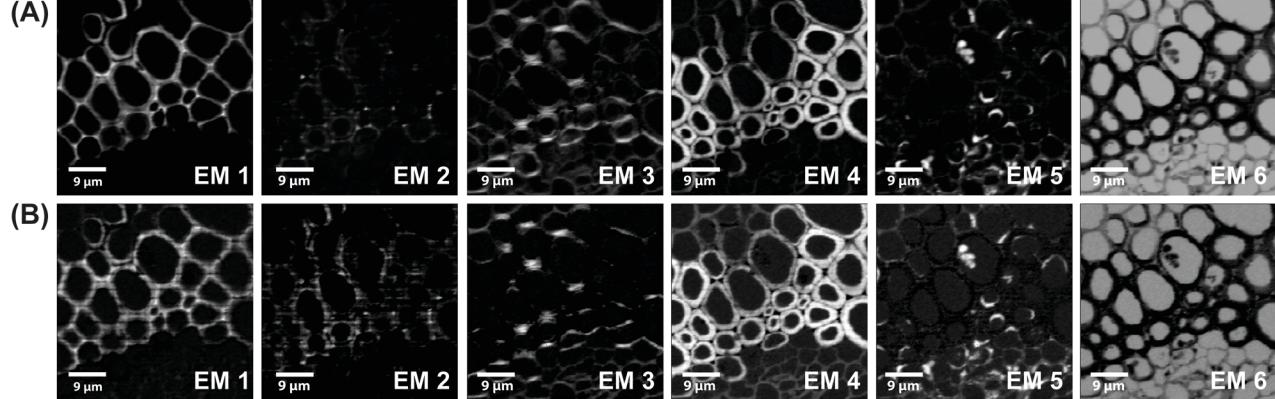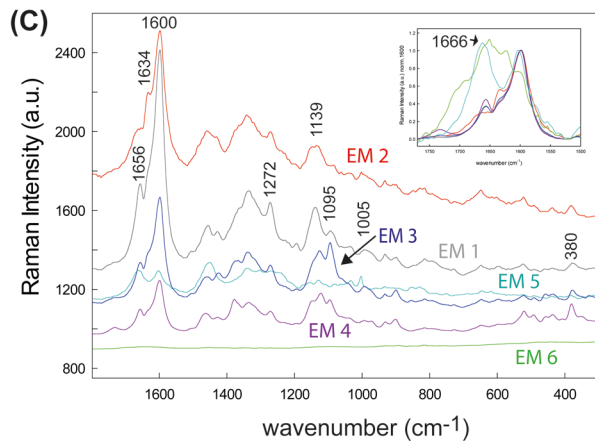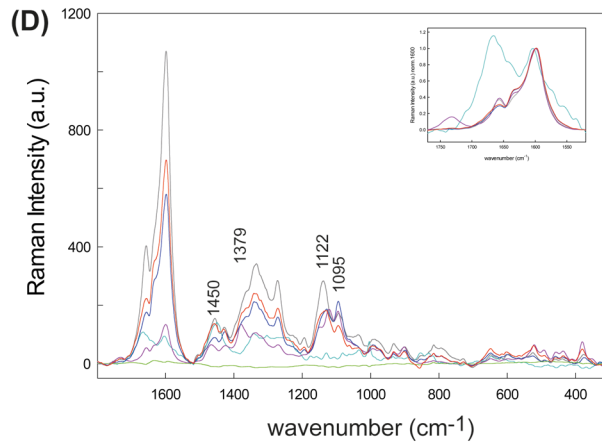

Supplement: Supplementary file 3 — Additional file 3: Figure S1. Influence of background subtraction on the Vertex Component Analysis with 6 endmembers on Arabidopsis thaliana. Intensity maps given by VCA on xylem and phloem of A. thaliana (A) without previous background (A) subtraction and (B) after background subtraction. Abundance maps are scaled equally two by two. The respective endmember spectra are shown in (C) and (D). [file 13007_2018_320_MOESM3_ESM.pdf]

EM 1

EM 2

(A)

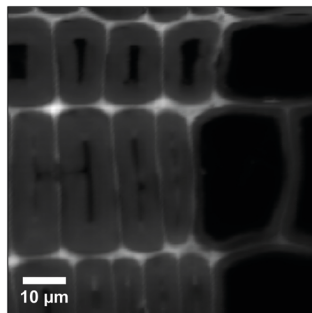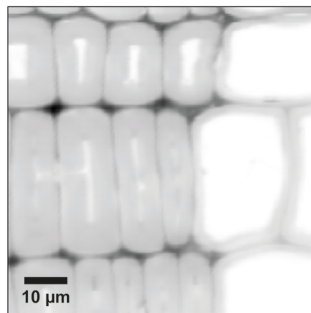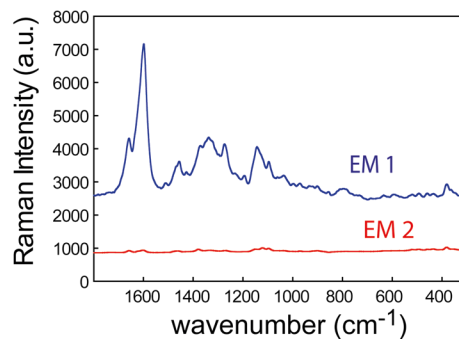

(B)

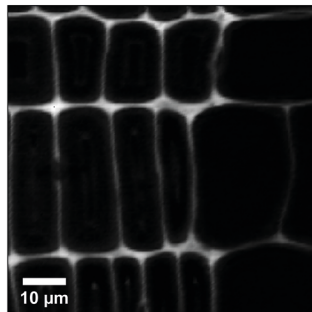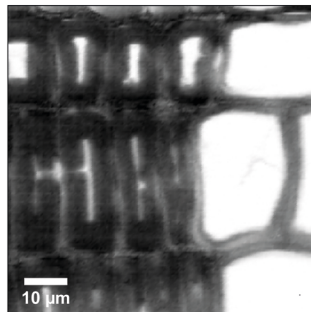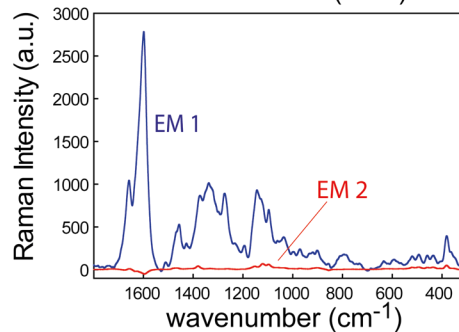

EM 1

EM 2

(A)

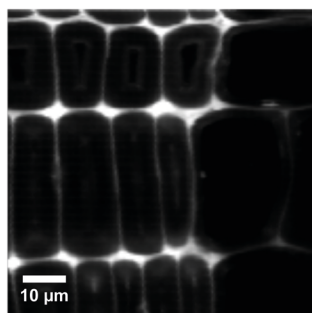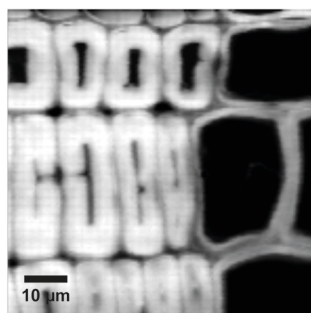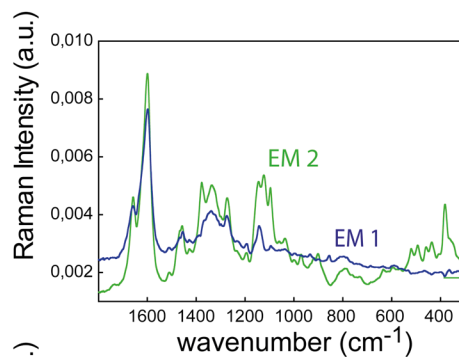

(B)

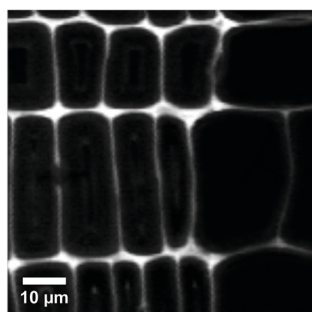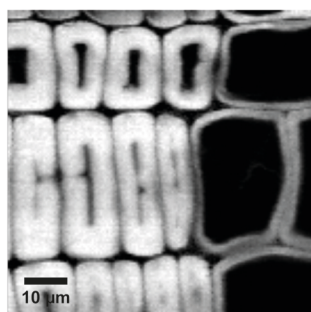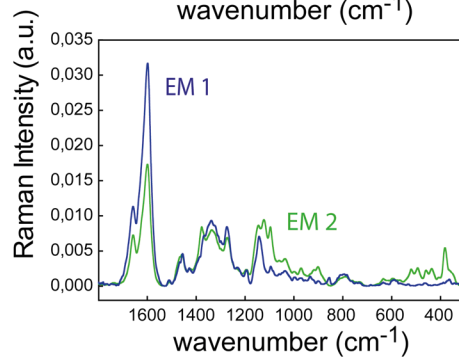

Supplement: Supplementary file 8 — Additional file 8: Figure S2. VCA (top) and NMF (bottom) analyses of the data set Spruce without previous background subtraction (A) and with background subtraction (B) with a rank of 2 endmembers. VCA is able to separate the plant material (EM1) and water (EM2) independently of the implementation of background correction. However, the cell wall is included in the water endmember when no baseline correction is applied. NMF by contrary deprecates the water component and the plant material is described by compound middle lamella (EM1) and cell wall (EM2). A rank of 2 is not enough to depict the main features of the plant tissue even after background correction, as NMF does. [file 13007_2018_320_MOESM8_ESM.pdf]

VCA

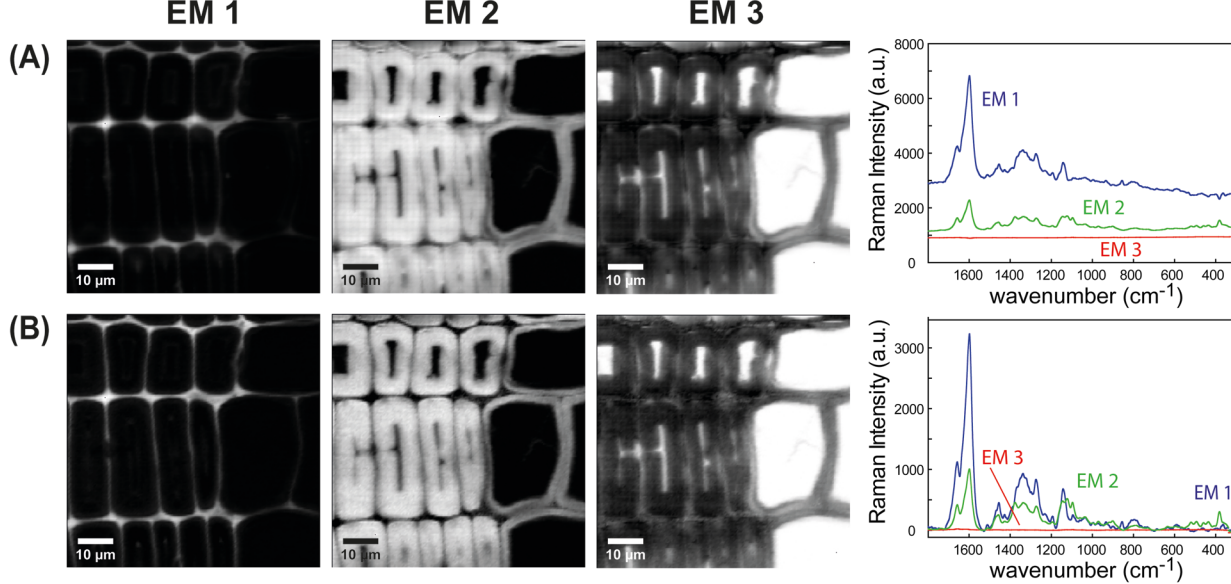

NMF

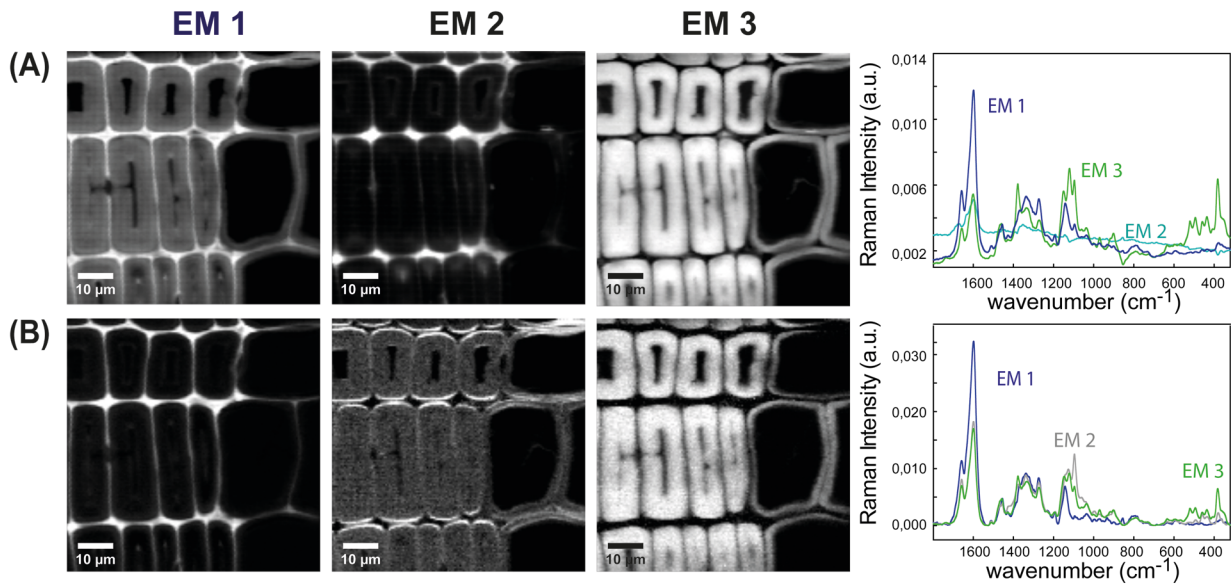

Supplement: Supplementary file 9 — Additional file 9: Figure S3. VCA (top) and NMF (bottom) analyses of the data set Spruce, without previous background subtraction (A) and with background subtraction (B) with a rank of 3 endmembers. [file 13007_2018_320_MOESM9_ESM.pdf]

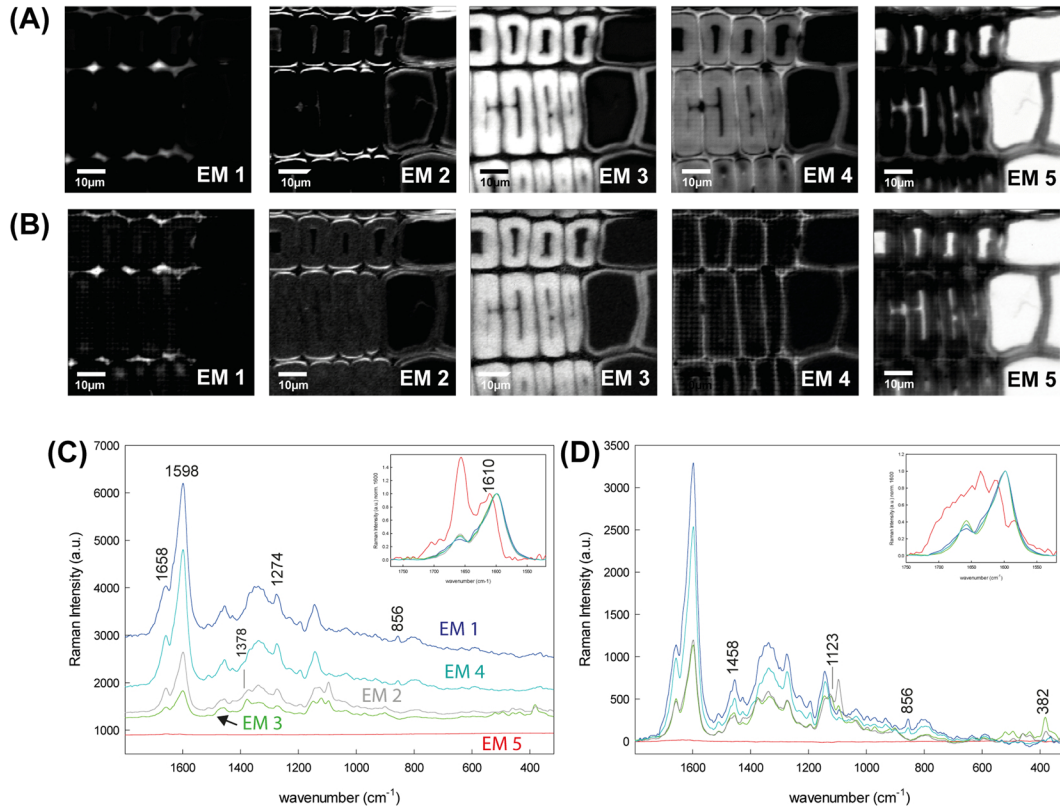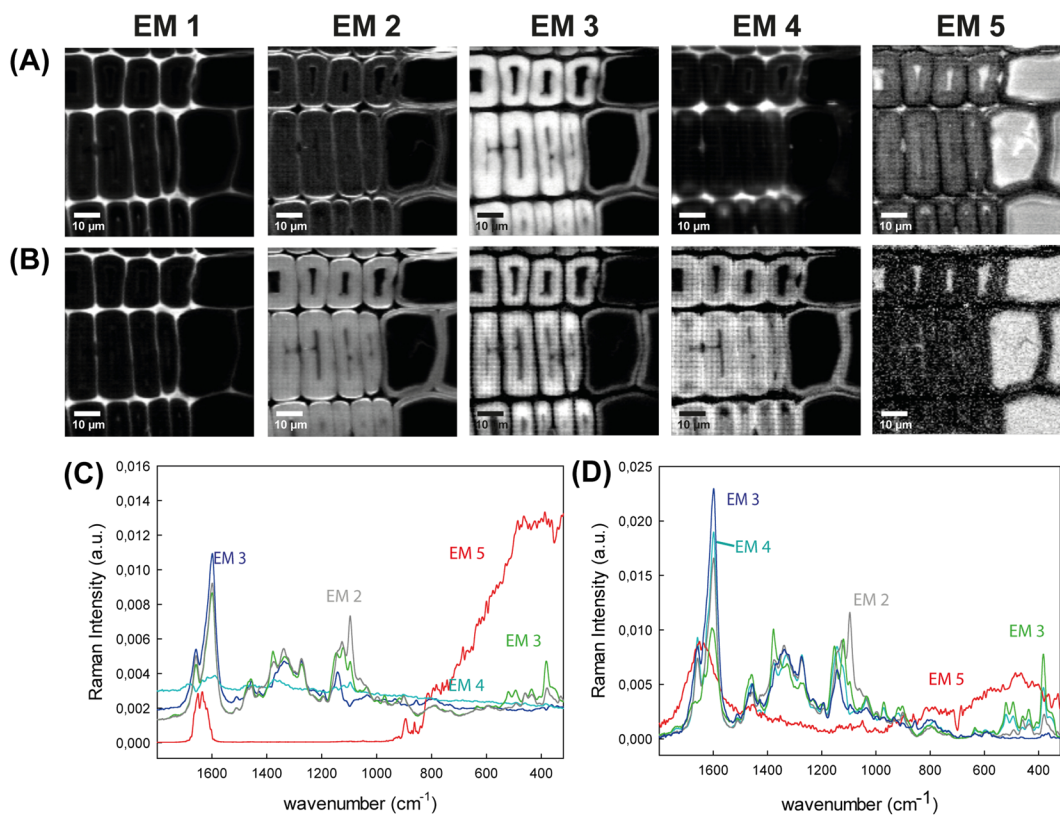

Supplement: Supplementary file 10 — Additional file 10: Figure S4. VCA (top) and NMF (bottom) analyses of the data set Spruce, without previous background subtraction (A) and with background subtraction (B) with a rank of 5 endmembers. For VCA (top), when no background subtraction is applied (A), EM1 shows the inner part of the cell corners whereas EM4 shows the outer cell corners but also part of the main cell wall. EM2 shows the cell wall layer S1 in which the cellulose microfibril orientation is parallel to the laser polarisation. Note the presence of pectin at 854 cm−1 in the cell corner in the EM1 and EM4. EM3 marks most of the cell wall (cellulose, hemicellulose and lignin) while EM5 is the lumen filled with water. (B) Distribution maps of the endmembers (EM) generated by VCA of Spruce with prior background subtraction. The abundance maps are similar distributed as (A) but for EM4, which does not incorporate parts of the cell wall but rather only the compound middle lamella. The intensity profiles of the same endmembers in A and B are equally scaled between same endmembers, having the brightest pixel the maximum intensity. (C) and (D) Corresponding characteristic endmember spectra of the abundance maps shown in (A) and (B), respectively. The main differences between EMs are attributed to the 1658 band (C=O stretching and C=C groups) (see also inserts) and the orientation of the cellulose microfibrils (bands at 1096, 1125 and lower spectral region 370–550 cm−1). [file 13007_2018_320_MOESM10_ESM.pdf]
