# Supplementary material for: Multivariate unmixing approaches on Raman images of plant cell walls: new insights or overinterpretation of results?
Source: Plant Methods. 2018 Jul 4;14:52. doi: 10.1186/s13007-018-0320-9 (PMC6031114; doi:10.1186/s13007-018-0320-9)
Supplement: Supplementary file 4 — Additional file 4: Table S3. Correlation coefficients between the spectral endmembers generated within each algorithm. (with 6 endmembers) for Arabidopsis without previous background subtraction. [file 13007_2018_320_MOESM4_ESM.docx]

**Table S3**

| Without BG subtraction | | | | | | | |
| --- | --- | --- | --- | --- | --- | --- | --- |
| VCA | | **NMF** | | **MCR no PCA** | | **MCR with PCA** | |
| EM | **r** | **EM** | **r** | **EM** | **r** | **EM** | **r** |
| 1-3 | 0.973 | 3-4 | 0.763 | 1-3 | 0.866 | 4-5 | 0.638 |
| 1-2 | 0.890 | 1-2 | 0.624 | 3-4 | 0.636 | 1-2 | 0.496 |
| 2-3 | 0.867 | 1-4 | 0.424 | 4-5 | 0.631 | 3-4 | 0.372 |
| 3-4 | 0.855 | 1-5 | 0.258 | 1-4 | 0.552 | 1-4 | 0.245 |
| 2-5 | 0.828 | 2-5 | 0.255 | 1-2 | 0.544 | 1-5 | 0.192 |
| 1-4 | 0.753 | 3-5 | 0.140 | 1-5 | 0.388 | 2-5 | 0.174 |
| 3-5 | 0.667 | 2-4 | 0.129 | 2-3 | 0.371 | 1-3 | 0.105 |
| 1-5 | 0.666 | 1-3 | 0.092 | 2-5 | 0.294 | 2-4 | 0.070 |
| 2-4 | 0.580 | 4-6 | 0.085 | 3-5 | 0.209 | 3-6 | 0.038 |
| 4-5 | 0.456 | 4-5 | 0.049 | 2-4 | 0.191 | 2-3 | 0.015 |
| 4-6 | -0.032 | 3-6 | -0.028 | 3-6 | -0.298 | 3-5 | -0.110 |
| 3-6 | -0.369 | 2-3 | -0.037 | 1-6 | -0.507 | 1-6 | -0.411 |
| 1-6 | -0.380 | 1-6 | -0.311 | 2-6 | -0.590 | 2-6 | -0.574 |
| 5-6 | -0.707 | 5-6 | -0.550 | 4-6 | -0.608 | 4-6 | -0.598 |
| 2-6 | -0.710 | 2-6 | -0.748 | 5-6 | -0.936 | 5-6 | -0.897 |
