# Supplementary material for: Multivariate unmixing approaches on Raman images of plant cell walls: new insights or overinterpretation of results?
Source: Plant Methods. 2018 Jul 4;14:52. doi: 10.1186/s13007-018-0320-9 (PMC6031114; doi:10.1186/s13007-018-0320-9)
Supplement: Supplementary file 6 — Additional file 6: Table S5. Correlation coefficients between the spectral endmembers generated by the algorithms (5 endmembers) for Spruce with previous background subtraction. The endmembers given by VCA were taken as reference for the comparison. [file 13007_2018_320_MOESM6_ESM.docx]

**Table S5**

|  | With BG subtraction | | | | | |
| --- | --- | --- | --- | --- | --- | --- |
| VCA |  | **EM 1** | **EM 2** | **EM 3** | **EM 4** | **EM 5** |
| EM 1 | VCA | 1.000 |  |  |  |  |
|  | NMF | 0.997 | 0.859 | 0.725 | 0.939 | 0.525 |
|  | MCR no PCA | 0.996 | 0.807 | 0.742 | 0.986 | 0.880 |
|  | MCR with PCA | 0.998 | 0.799 | 0.717 | 0.503 | 0.881 |
| EM 2 | VCA |  | 1 |  |  |  |
|  | NMF | 0.908 | 0.994 | 0.817 | 0.911 | 0.348 |
|  | MCR no PCA | 0.908 | 0.981 | 0.885 | 0.856 | 0.908 |
|  | MCR with PCA | 0.897 | 0.977 | 0.868 | 0.641 | 0.914 |
| EM 3 | VCA |  |  | 1.000 |  |  |
|  | NMF | 0.927 | 0.960 | 0.892 | 0.977 | 0.364 |
|  | MCR no PCA | 0.930 | 0.923 | 0.923 | 0.969 | 0.864 |
|  | MCR with PCA | 0.913 | 0.913 | 0.887 | 0.704 | 0.938 |
| EM 4 | VCA |  |  |  | 1.000 |  |
|  | NMF | 0.998 | 0.874 | 0.722 | 0.953 | 0.513 |
|  | MCR no PCA | 0.998 | 0.819 | 0.745 | 0.914 | 0.975 |
|  | MCR with PCA | 0.995 | 0.810 | 0.714 | 0.507 | 0.912 |
| EM 5 | VCA |  |  |  |  | 1.000 |
|  | NMF | 0.523 | 0.506 | 0.338 | 0.428 | 0.651 |
|  | MCR no PCA | 0.514 | 0.508 | 0.409 | 0.445 | 0.530 |
|  | MCR with PCA | 0.536 | 0.527 | 0.499 | -0.070 | 0.391 |
